# Elimination of Mutant mtDNA by an Optimized mpTALEN Restores Differentiation Capacities of Heteroplasmic MELAS-iPSCs

**DOI:** 10.1016/j.omtm.2020.10.017

**Published:** 2020-10-22

**Authors:** Naoki Yahata, Hiroko Boda, Ryuji Hata

**Affiliations:** 1 Department of Anatomy I, Fujita Health University School of Medicine, Toyoake, Aichi 470-1192, Japan; 2 Department of Pediatrics, Fujita Health University School of Medicine, Toyoake, Aichi 470-1192, Japan

**Keywords:** disease modeling, induced pluripotent stem cell, mitochondrial DNA, myocyte, TALEN

## Abstract

Various mitochondrial diseases, including mitochondrial encephalopathy, lactic acidosis, and stroke-like episodes (MELAS), are associated with heteroplasmic mutations in mitochondrial DNA (mtDNA). Herein, we refined a previously generated G13513A mtDNA-targeted platinum transcription activator-like effector nuclease (G13513A-mpTALEN) to more efficiently manipulate mtDNA heteroplasmy in MELAS-induced pluripotent stem cells (iPSCs). Introduction of a nonconventional TALE array at position 6 in the mpTALEN monomer, which recognizes the sequence around the m.13513G>A position, improved the mpTALEN effect on the heteroplasmic shift. Furthermore, the reduced expression of the new Lv-mpTALEN(PKLB)/R-mpTALEN(PKR6C) pair by modifying codons in their expression vectors could suppress the reduction in the mtDNA copy number, which contributed to the rapid recovery of mtDNA in mpTALEN-applied iPSCs during subsequent culturing. Moreover, MELAS-iPSCs with a high proportion of G13513A mutant mtDNA showed unusual properties of spontaneous, embryoid body-mediated differentiation *in vitro*, which was relieved by decreasing the heteroplasmy level with G13513A-mpTALEN. Additionally, drug-inducible, myogenic differentiation 1 (MYOD)-transfected MELAS-iPSCs (MyoD-iPSCs) efficiently differentiated into myosin heavy chain-positive myocytes, with or without mutant mtDNA. Hence, heteroplasmic MyoD-iPSCs controlled by fine-tuned mpTALENs may contribute to a detailed analysis of the relationship between mutation load and cellular phenotypes in disease modeling.

## Graphical Abstract

**Figure:**
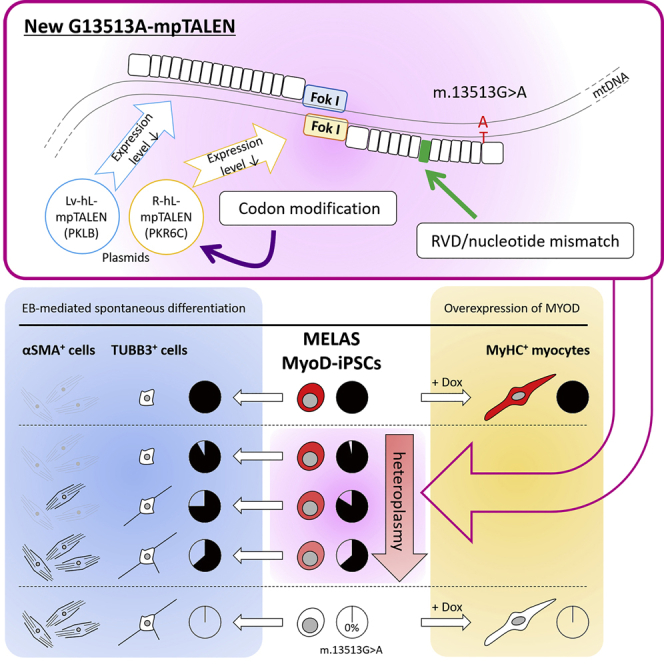

## Teaser

Yahata et al. refined a previous G13513A-mpTALEN to more efficiently manipulate mtDNA heteroplasmy in MELAS-iPSCs. Mutation-rich MELAS-iPSCs showed unusual differentiation properties, which were recovered by decreasing the heteroplasmy levels with new G13513A-mpTALEN. They further demonstrated that drug-inducible, MYOD-transfected MELAS-iPSCs could efficiently differentiate into MyHC-positive myocytes, with or without mutant mtDNA.

## Introduction

Mitochondrial diseases are a heterogeneous group of multisystem disorders caused by mitochondrial dysfunction, including defects in the respiratory chain/oxidative phosphorylation system. A portion of these diseases are caused by various point mutations in mitochondrial DNA (mtDNA).^1^ However, patients with these diseases can contain both normal and mutant mtDNA in an individual cell, a phenomenon termed heteroplasmy. The degree of heteroplasmy, and the distribution of mutant mtDNA in patient tissues, determines the severity and phenotypic heterogeneity of these diseases. Therefore, to effectively study the pathogenesis and potential therapies for these diseases, it is necessary to develop a means by which the degree of mtDNA heteroplasmy can be manipulated.

Recent advances in programmable nucleases, such as zinc finger nuclease (ZFN),^2^ transcription activator-like effector nuclease (TALEN),^3^, ^4^, ^5^, ^6^ and mitoTev-TALE,^7^ allow for the controlled alteration of mtDNA heteroplasmy in patient cells and cybrids. In fact, we have previously engineered an mtDNA-targeted platinum TALEN (mpTALEN) capable of preferentially cleaving mtDNA carrying the G13513A mutation,^8^ which is known to cause mitochondrial encephalopathy, lactic acidosis, and stroke-like episodes (MELAS), and/or Leigh syndrome.^9^ We have also demonstrated that the heteroplasmy level in MELAS-induced pluripotent stem cells (iPSCs) harboring the m.13513G>A mutation decreased within a short period following administration of G13513A-mpTALEN.^8^ A previous report using a mitochondrial-targeted ZFN (mtZFN) for the m.8993T>G mutation demonstrated that finely controlled expression of mtZFN not only limited an undesired loss in mtDNA copy numbers, but it also increased the effectiveness of mtDNA heteroplasmy shifting in cybrid cells.^10^

To date, disease-specific iPSCs have been established for mitochondrial diseases caused by various mtDNA mutations, including m.13513G>A.^11^, ^12^, ^13^ However, various studies have reported that patient-derived iPSCs with a relatively high percentage of mutant mtDNA cannot differentiate into various types of targeted cells. For instance, iPSCs from patients with Leigh syndrome harboring the m.13513G>A (>35% heteroplasmy) or m.8993T>G (∼100%) mutation were unable to differentiate into cardiac troponin T-positive cells, showing spontaneous beating activity.^13^ Additionally, patient-derived iPSCs with high proportions of the m.3243A>G mutation (≥90%) were associated with neuronal and cardiac maturation defects.^14^ Similarly, iPSCs derived from patients with MELAS, who had ∼100% m.5541C>T mtDNA, showed a significant loss of ability to terminally differentiate into neurons.^15^

Meanwhile, few reports are available regarding skeletal muscle cells differentiated from patient-derived iPSCs with mutant mtDNA.^11^ Therefore, to better understand why some patients with MELAS and/or Leigh syndrome carrying the m.13513G>A mutation show muscular atrophy,^16^ ragged red fibers, and decreased activities of mitochondrial respiratory complexes in muscle biopsy samples,^9^^,^^17^ more sophisticated models capable of recapitulating the cellular phenotypes occurring in mitochondrial diseases caused by point mutations in mtDNA are required.

To this end, in the present study we aimed to develop a more effective G13513A-mpTALEN that enhances the heteroplasmic shift of mtDNA in MELAS-iPSCs with m.13513G>A mutation. We also sought to demonstrate the relationship between differentiation properties and the proportion of mutant mtDNA in isogenic iPSCs using renewed G13513A-mpTALEN. Lastly, we describe the efficient generation of one type of targeted cells, namely myocytes, from MELAS-iPSCs, with or without mutant mtDNA, in a short period. This study, therefore, presents a research strategy for heteroplasmic iPSC modeling of mitochondrial diseases with point-mutated mtDNA.

## Results

### Refinement of G13513A-mpTALEN

**Figure 1:**
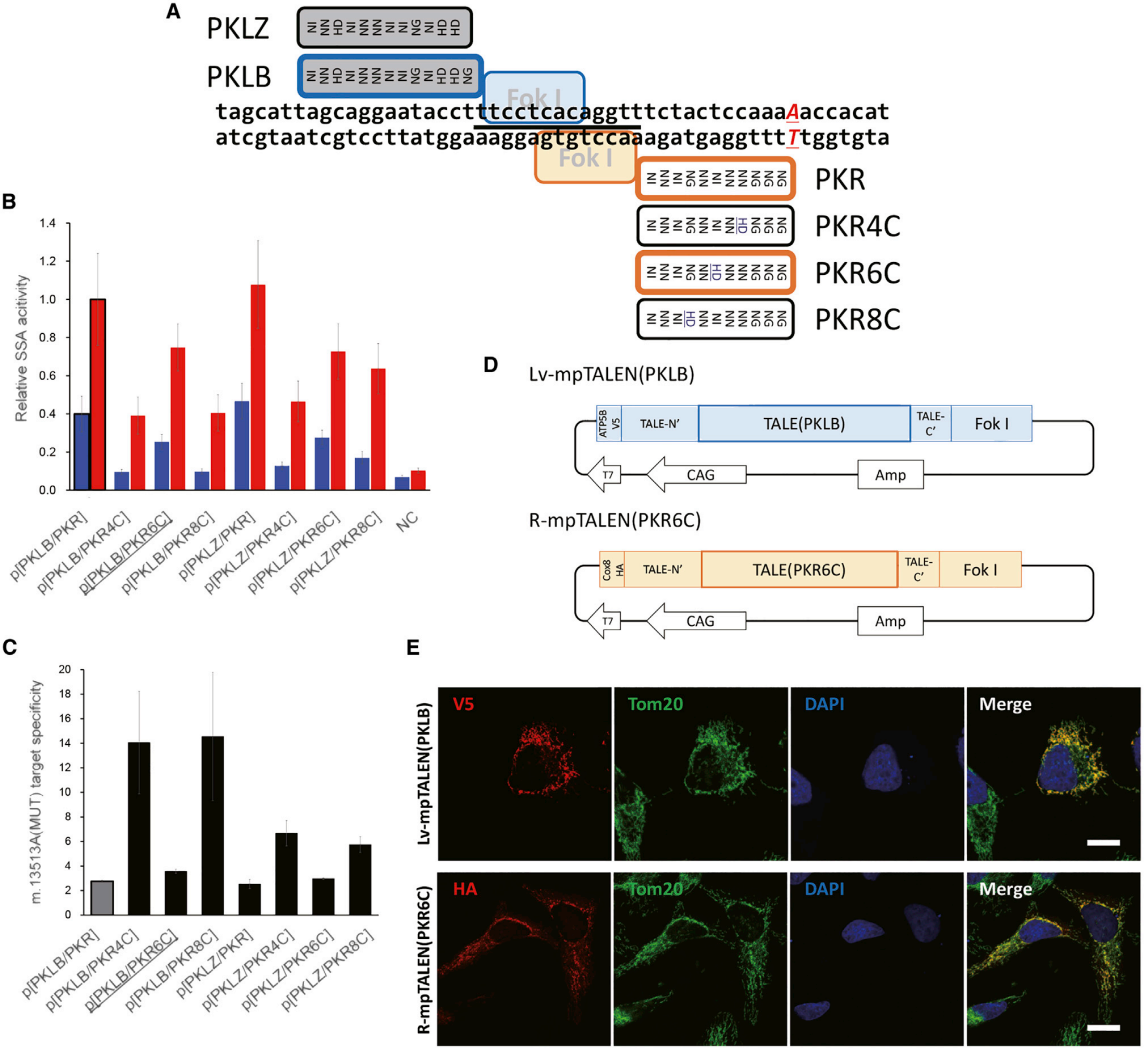
Refinement of G13513A-mpTALEN (A) Schematic illustration of the target site of G13513A-pTALEN. Gray and white boxes indicate RVDs in left-pTALENs and right-pTALENs, respectively. Names of TALEs are indicated next to the boxes. The spacer region of the pTALEN(PKLB)/pTALEN(PKR) pair (abbreviated as p[PKLB/PKR]) is underlined. (B) Evaluation of the SSA activity (Luc/RLuc) of pTALEN pairs. Blue and red bars indicate the cleaving activity against human mtDNA sequences, including m.13513G(WT) and m.13513A(MUT), respectively. Relative SSA activity is defined as the ratio of the measured activity to the activity score of the p[PKLB/PKR]. Data are expressed as the mean ± SEM (n = 3). NC, negative control. (C) Specificity of each left-pTALEN/right-pTALEN pair toward the m.13513A(MUT) target. Data are expressed as the mean ± SEM (n = 3). (D) Components of plasmids used to express Lv-mpTALEN(PKLB) and R-mpTALEN(PKR6C) monomers. ATP5B and Cox8, mitochondrial targeting sequences of *ATP5B* and *Cox8*; V5, V5-tag; HA, HA-tag; T7, T7 promoter; CAG, CAG promoter; Amp, ampicillin resistance gene. (E) Intracellular localization of mpTALENs analyzed by immunocytochemistry. Lv-mpTALEN(PKLB) and R-mpTALEN(PKR6C) were transiently expressed in HeLa cells and then stained with an anti-V5 or anti-HA antibody, respectively (red), at 2 days after the transfection. Mitochondria were stained with an anti-TOM20 antibody (green). Nuclei were stained with DAPI (blue). Scale bars, 20 μm.

To develop a more effective G13513A-mpTALEN, various types of pTALEN pairs were designed. TALENs contain the *Fok*I nuclease domain and a DNA binding domain (TALE), which is composed of tandem arrays of a 34-aa repeat module. We used a conventional DNA recognition code in the TALE domain, repeat variable di-residues (RVDs), NI, HD, NN, and NG, which recognize adenine (A), cytosine (C), guanine (G), and thymine (T), respectively. Immediately upstream of the DNA sequence, recognized by RVDs of the pTALEN monomer (position 0), was “T” (Figures 1A and S1B–S1D). A previously reported G13513A-mpTALEN pair, L-mpTALEN(PKLB) and R-mpTALEN(PKR),^8^ had 13 and 11 RVDs, respectively (Figure 1A), with the R-mpTALEN(PKR) monomer recognizing the m.13513G>A position. We newly designed three types of right-pTALEN monomers having 11 RVDs to recognize the m.13513G>A position (names of TALEs: PNR, POR, and PQR; Figures S1B and S1C). We additionally designed two right-pTALENs, named pTALEN(PKRY) and pTALEN(PKRX), in which one and two modules were removed from pTALEN(PKR), respectively (Figure S1D). In contrast, the L-mpTALEN(PKLB) monomer did not directly interact with the m.13513G>A position. We also prepared several left-pTALENs (names of TALEs: PKLA, PKLZ, PKLC, PKLD, PKLE, PHL, and PIL; Figures 1A and S1B–S1D), in which several modules were added to or removed from pTALEN(PKLB). Other types of left-pTALENs, namely, pTALEN(PMTL) and pTALEN(PFR), were also prepared (Figure S1C). To examine the activity and specificity of the designed G13513A-pTALENs, we performed a mammalian cell-based single-strand annealing (SSA) assay^18^ using a reporter plasmid carrying a fragment of the mtDNA sequence, including either m.13513G(WT) or m.13513A(MUT)^8^ (Figure S1A). Several G13513A-pTALEN pairs, including pTALEN(PNR), pTALEN(POR), or pTALEN(PQR), showed a higher mtDNA-cleaving activity than did the pTALEN(PKLB)/pTALEN(PKR) pair (abbreviated as p[PKLB/PKR]) but with a lower m.13513A(MUT) target specificity (Figure S1B). Meanwhile, other G13513A-pTALEN pairs, including pTALEN(PKRX) or pTALEN(PKRY), showed a higher m.13513A(MUT) target specificity than did p[PKLB/PKR] but a much lower mtDNA-cleaving activity (Figure S1D).

A previous report has indicated that the TALEN can only accommodate a relatively small number of position-dependent mismatches between conventional RVDs and nucleotides.^19^ We tried to introduce an RVD/nucleotide mismatch into the TALE array of G13513A-mpTALEN to increase the target specificity for G13513A mutant mtDNA. The RVD at positions 4, 6, or 8 of the right-pTALEN(PKR) monomer was changed from NN, NI, or NG to HD, resulting in pTALEN(PKR4C), pTALEN(PKR6C), or pTALEN(PKR8C), respectively (Figure 1A). All possible combinations of two left-pTALENs [pTALEN(PKLB) and pTALEN(PKLZ)] and four right-pTALENs [pTALEN(PKR), pTALEN(PKR4C), pTALEN(PKR6C), and pTALEN(PKR8C)] were evaluated using the SSA assay (Figures 1B and 1C). The p[PKLB/PKR4C] and p[PKLB/PKR8C] showed higher m.13513A(MUT) target specificities than did the other pairs (Figure 1C), but their cleaving activities were low (Figure 1B). Our previous study showed that the effect of the L-mpTALEN(PKLB)/R-mpTALEN(PKR4C) pair (abbreviated as mp[PKLB/PKR4C]; abbreviations for mpTALEN pairs are listed in Table 1) on mtDNA heteroplasmy in MELAS-iPSCs was lower than that of the mp[PKLB/PKR] due to insufficient mtDNA-cleaving activity of the former.^8^ Therefore, we focused on the p[PKLB/PKR6C] and p[PKLZ/PKR8C], which showed somewhat higher m.13513A(MUT) target specificities and somewhat lower cleaving activities than did the p[PKLB/PKR], respectively (Figures 1B and 1C).

**Table 1:** List of Abbreviations for mpTALEN Pairs

Abbreviation	Left-mpTALEN	Right-mpTALEN
mp[PKLB/PKR]	L-mpTALEN(PKLB)	R-mpTALEN(PKR)
mp[vPKLB/PKR]	Lv-mpTALEN(PKLB)	R-mpTALEN(PKR)
mp[PKLB/PKR6C]	L-mpTALEN(PKLB)	R-mpTALEN(PKR6C)
mp[vPKLB/PKR6C]	Lv-mpTALEN(PKLB)	R-mpTALEN(PKR6C)
mp[PKLB/PKR4C]	L-mpTALEN(PKLB)	R-mpTALEN(PKR4C)
mp[vPKLZ/PKR8C]	Lv-mpTALEN(PKLZ)	R-mpTALEN(PKR8C)
hL-mp[vPKLB/PKR]	Lv-hL-mpTALEN(PKLB)	R-hL-mpTALEN(PKR)
hL-mp[vPKLB/PKR6C]	Lv-hL-mpTALEN(PKLB)	R-hL-mpTALEN(PKR6C)
hL2-mp[vPKLB/PKR6C]	Lv-hL2-mpTALEN(PKLB)	R-hL2-mpTALEN(PKR6C)
hL2-mp[vPKLBm/PKR6Cm]	Lv-hL2-mpTALEN(PKLB)mut	R-hL2-mpTALEN(PKR6C)mut

### Validation of New G13513A-mpTALENs in MELAS-iPSCs

The two pTALEN pairs, p[PKLB/PKR6C] and p[PKLZ/PKR8C], selected as promising candidates for improved G13513A-pTALEN, were modified for mitochondrial localization and function (mpTALEN). As previously reported, R-mpTALEN or L-mpTALEN had a mitochondrial targeting sequence (MTS) of *cytochrome* *c* *oxidase subunit 8A* (*COX8*) or *ATP synthase* *F1* *subunit* *beta* (*ATP5B*) and an epitope tag, hemagglutinin (HA) or FLAG, at the N terminus, respectively.^8^ Additionally, we prepared another type of the left-mpTALEN monomer, Lv-mpTALEN, which possessed an MTS of *ATP5B* and a V5-tag^20^ at the N terminus (Figure 1D). To examine whether mpTALENs are readily imported into mitochondria, we analyzed their intracellular localization in transiently expressing HeLa cells. Immunofluorescence analysis revealed that mpTALENs colocalized with the mitochondrial marker TOM20 and were not observed in the nucleus (Figures 1E and S2).

**Figure 2:**
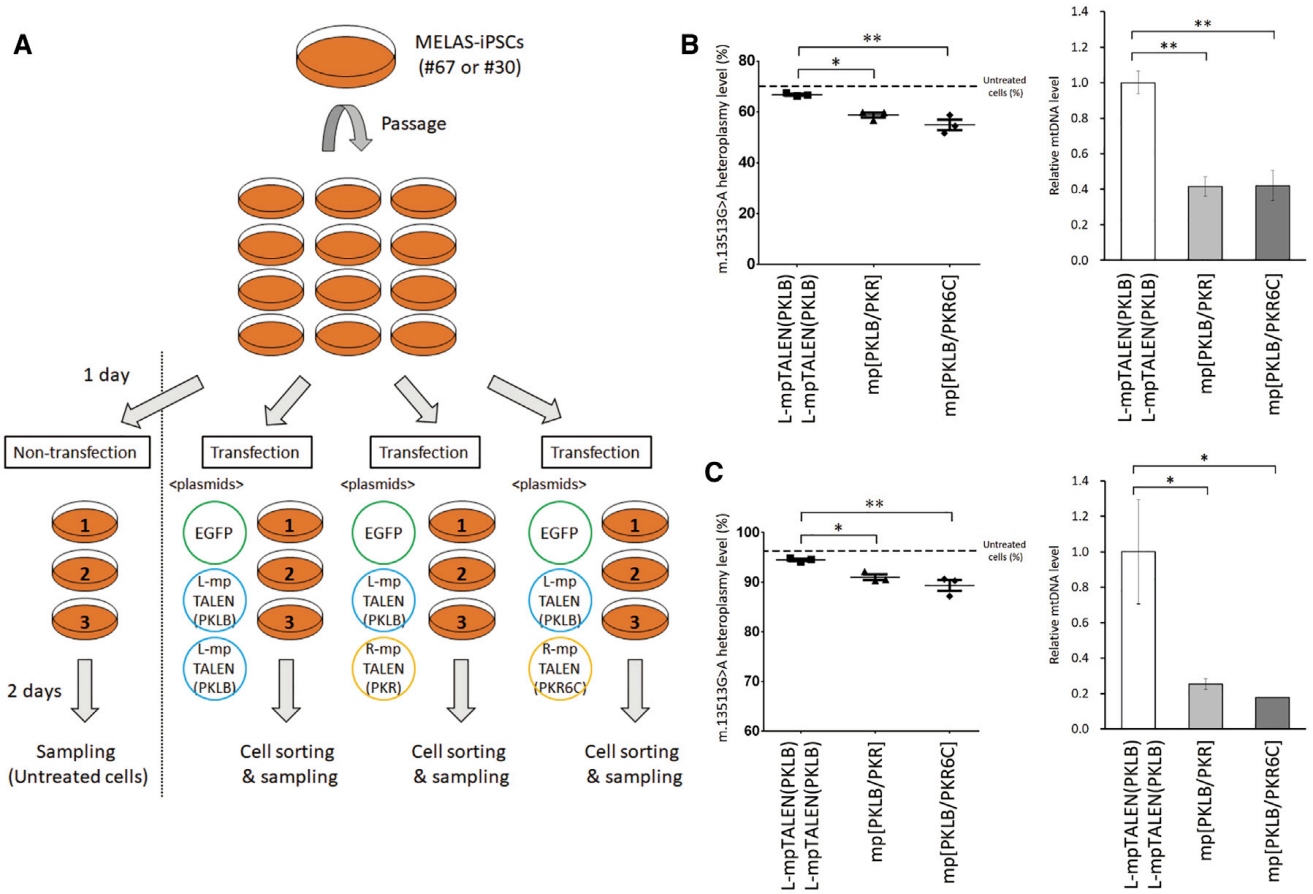
Effect of the mp[PKLB/PKR6C] on the Heteroplasmy Level in MELAS-iPSCs (A) Experimental scheme for the application of G13513A-mpTALEN to MELAS-iPSCs. MELAS-iPSCs (A01 #67 or #30) were transfected with plasmids coding mpTALENs and EGFP. EGFP-positive and live cells were analyzed on day 2 after the transfection. The effects of mp[PKLB/PKR6C] expression on the mtDNA heteroplasmy and copy numbers in MELAS-iPSCs were compared with those of mp[PKLB/PKR] expression and L-mpTALEN(PKLB) expression (transfected at twice the amount). (B and C, left) m.13513G>A heteroplasmy levels in MELAS-iPSCs (#67 and #30) were analyzed using ARMS-qPCR. Dotted line indicates the heteroplasmy level in untreated cells. Data are expressed as the mean ± SEM (n = 3). ∗p < 0.05, ∗∗p < 0.01 (Tukey’s test). (B and C, right) mtDNA copy numbers in MELAS-iPSCs (#67 and #30). Data are presented relative to those of L-mpTALEN(PKLB)/L-mpTALEN(PKLB) and expressed as the mean ± SEM (n = 3). ∗p < 0.05, ∗∗p < 0.01 (Tukey’s test).

We next evaluated the efficacy of the two G13513A-mpTALEN pairs in altering the mtDNA heteroplasmy levels in MELAS-iPSCs. The L-mpTALEN(PKLB)/R-mpTALEN(PKR6C) or L-mpTALEN(PKLB)/R-mpTALEN(PKR) plasmids were introduced into previously established A01 #67 or #30 MELAS-iPSCs,^8^ which were cultured under feeder-free conditions. EGFP was coexpressed as a transfection marker. On day 2 after the transfection, EGFP-positive and live (propidium iodide [PI]-negative) cells were sorted using a cell sorter. The m.13513G>A heteroplasmy levels in the sorted cells were analyzed using allele refractory mutation system-based quantitative PCR (ARMS-qPCR) and compared with that in sorted cells that were transfected with twice the amount of the L-mpTALEN(PKLB) plasmid so as not to change the total concentration of the plasmid and the expression levels of unnatural proteins (Figure 2A). The data showed that the expression of mp[PKLB/PKR] and mp[PKLB/PKR6C] decreased the m.13513G>A heteroplasmy levels in MELAS-iPSCs (#67 and #30) compared with that in the cells transfected with L-mpTALEN(PKLB) only. The mp[PKLB/PKR6C] showed the greatest reduction in the percentage of mutant mtDNA (Figures 2B and 2C). After applying L-mpTALEN(PKLB) only or the mp[PKLB/PKR6C] for 2 days, the m.13513G>A heteroplasmy levels were 66.8% or 55.0% in #67 iPSCs and 94.5% or 89.4% in #30 iPSCs, respectively (Figures 2B and 2C). These data indicated that the diminution rate of mutant mtDNA by G13513A-mpTALEN during the same period (∼12%, #67 iPSCs; ∼5%, #30 iPSCs) is dependent on the heteroplasmy level before mpTALEN application. A loss of the mtDNA copy number, due to the nuclease activity of G13513A-mpTALEN, was observed (Figures 2B and 2C). In contrast, the application of the mp[vPKLZ/PKR8C] for 2 days could not induce any significant change in mtDNA heteroplasmy compared with that in the cells transfected with Lv-mpTALEN(PKLZ) only (Figure S3). Thus, the mp[PKLB/PKR6C] showed the best performance in terms of decreasing the percentage of G13513A mtDNA in heteroplasmic iPSCs at this point.

### Effects of the G13513A-mpTALEN Concentration on Heteroplasmic mtDNA Profiles

**Figure 3:**
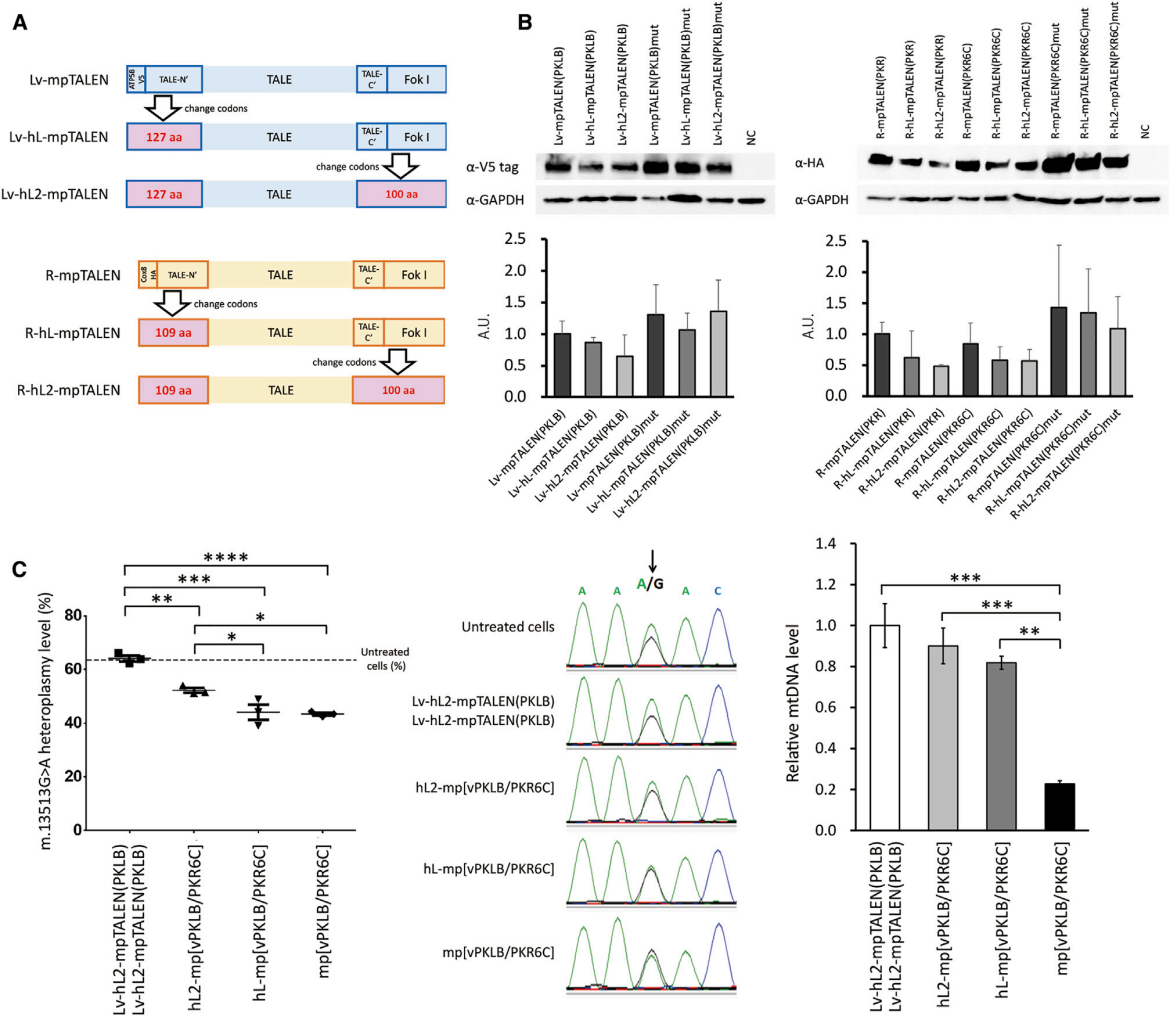
Effects of G13513A-mpTALEN Expression Levels on mtDNA Heteroplasmy and the Depletion of mtDNA Copy Numbers in MELAS-iPSCs (A) Schematic illustration of codon modification in the Lv-mpTALEN and R-mpTALEN genes. The codons of N-terminal 127 and 109 aa in the Lv-mpTALEN and R-mpTALEN genes were altered into selected codons (listed in Figure S4C), showing a lower frequency of usage in humans (*hL*) (resulting in Lv-*hL*-mpTALEN and R-hL-mpTALEN, respectively). Furthermore, the codons of C-terminal 100 aa in the Lv-hL-mpTALEN and R-hL-mpTALEN genes were also altered into selected codons (resulting in Lv-hL2-mpTALEN and R-hL2-mpTALEN, respectively). (B) Upper: representative western blotting results for HEK293T cells transfected with mpTALEN plasmids for 2 days. The Lv-mpTALEN and R-mpTALEN proteins were detected using anti-V5 and anti-HA antibodies, respectively. GAPDH served as a loading control. Lower: expression levels were first quantified by normalization of the mpTALEN signal to that of GAPDH and, subsequently, by calculating the ratio with respect to the mean value of the normalized Lv-mpTALEN(PKLB) or R-mpTALEN(PKR) signal, respectively (n = 3; error bars, SD). (C) Effects of mp[vPKLB/PKR6C] expression levels on m.13513G>A heteroplasmy and mtDNA copy numbers in MELAS-iPSCs (#67). Sorted cells were analyzed 2 days after the transfection. Left: ARMS-qPCR data. Dotted line indicates the heteroplasmy level in untreated cells. Data are expressed as the mean ± SEM (n = 3). ∗p < 0.05, ∗∗p < 0.01, ∗∗∗p < 0.001, ∗∗∗∗p < 0.0001 (Tukey’s test). Middle: Sanger sequencing data. Arrow indicates the m.13513 position. Right: mtDNA copy numbers are presented relative to those in cells transfected with Lv-hL2-mpTALEN(PKLB)/Lv-hL2-mpTALEN(PKLB). Data are expressed as the mean ± SEM (n = 3). ∗∗p < 0.01, ∗∗∗p < 0.001 (Tukey’s test).

We next assessed the influence of the mpTALEN expression level on mtDNA heteroplasmy and copy numbers in MELAS-iPSCs. To decrease the mpTALEN expression level in human cells, codons of the Lv-mpTALEN and R-mpTALEN genes were changed in their expression plasmids, based on the information regarding codon usage bias in *Homo sapiens* from an international DNA sequence database.^21^ First, 127 and 109 higher-usage codons in the N-terminal regions of Lv- and R-mpTALENs, including the MTS and epitope tag, were changed to low-usage codons, and the resulting TALENs were named Lv-hL-mpTALENs and R-hL-mpTALENs, respectively (Figures 3A and S4). Additionally, 100 higher-usage codons in the C-terminal region of mpTALEN, including the *Fok*I domain, were also changed to low-usage codons, and the resulting TALEN was named hL2-mpTALEN (Figures 3A and S4). We did not modify codons in the TALE domain of mpTALEN to be able to maintain the Golden Gate assembly system for the construction of TALEN vectors. The expression levels of mpTALENs, hL-mpTALENs, and hL2-mpTALENs were compared in HEK293T cells by western blotting (Figure 3B). The expression level of each mpTALEN showed a downward trend, according to stepwise changes in the codons. The expression level of hL2-mpTALEN was approximately half that of the original mpTALEN (Figure 3B).

We further analyzed the effects of the expression levels of the most promising mp[vPKLB/PKR6C] on the m.13513G>A mtDNA heteroplasmy levels and copy numbers in #67 iPSCs (Figure 3C). The heteroplasmy levels in sorted cells expressing mp[vPKLB/PKR6C], hL-mp[vPKLB/PKR6C], hL2-mp[vPKLB/PKR6C], and Lv-hL2-mpTALEN(PKLB) only were 43.4%, 44.1%, 52.2%, and 64.1%, respectively (Figure 3C). The sequencing data showed a similar trend in mtDNA heteroplasmy (Figure 3C). The mp[vPKLB/PKR6C] induced the highest reduction in the percentage of mutant mtDNA among the three pairs (Figure 3C). The mtDNA copy numbers decreased in sorted cells expressing mp[vPKLB/PKR6C], hL-mp[vPKLB/PKR6C], and hL2- mp[vPKLB/PKR6C] to 23%, 82%, and 90% of that in sorted cells expressing Lv-hL2-mpTALEN(PKLB) only, respectively (Figure 3C). These data indicated that the mtDNA depletion caused by the application of the hL-mp[vPKLB/PKR6C] was obviously lower than that caused by the application of the mp[vPKLB/PKR6C]. Two additional experiments showed a similar tendency (Figure S5). Furthermore, the application of the hL-mp[vPKLB/PKR] resulted in comparable effects on mtDNA profiles (Figure S6). Thus, the effect of the hL2-mp[vPKLB/PKR6C] on mtDNA heteroplasmy was the lowest among the three pairs tested and the depletion of the mtDNA copy number was the mildest (Figure 3C). These data suggested that codon changes in the N-terminal sequence of mpTALEN drastically suppressed the reduction in the mtDNA copy number in MELAS-iPSCs.

### Long-Term Effects of G13513A-mpTALEN Application on Heteroplasmic iPSCs

**Figure 4:**
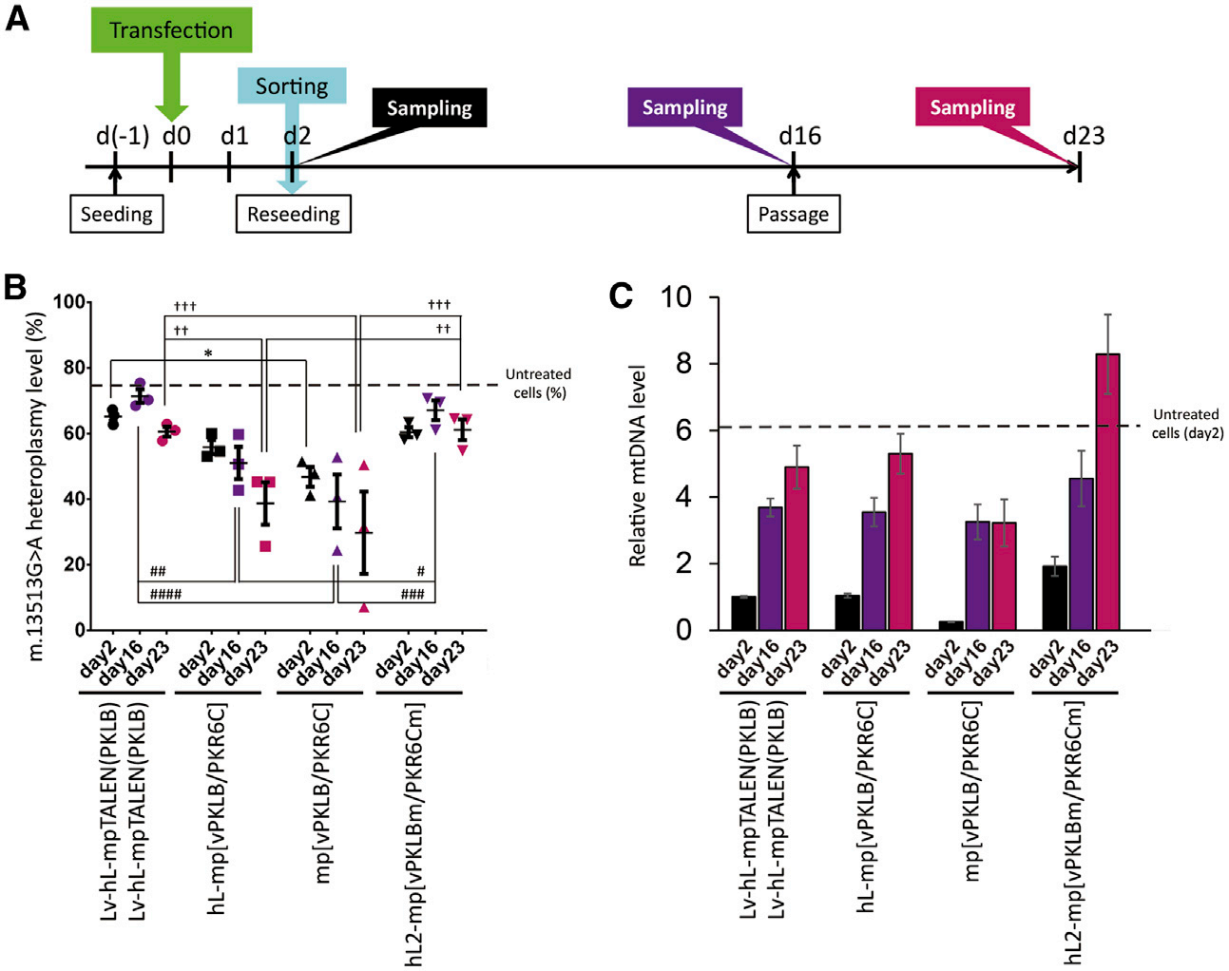
Long-Term Effects of Transient G13513A-mpTALEN Expression on mtDNA Heteroplasmy and Copy Numbers in MELAS-iPSCs (A) Experimental scheme. Two days after transfection, sorted cells were re-cultured without feeder cells for 21 days. (B) Heteroplasmy levels at 2, 16, and 23 days after transfection. Data are expressed as the mean ± SEM (n = 3). Dotted line indicates the heteroplasmy level in untreated cells on day 2. ∗^, #^p < 0.05, ^##,††^p < 0.01, ^###,†††^p < 0.001, ^####^p < 0.0001 (one-way ANOVA, followed by Tukey’s test). (C) mtDNA copy numbers at 2, 16, and 23 days after the transfection. Data are presented as the ratio of the measured number to that in cells transfected with Lv-hL-mpTALEN(PKLB) only, at 2 days after the transfection. Dotted line indicates the relative copy number in untreated cells on day 2. Data are expressed as the mean ± SEM (n = 3).

We monitored mtDNA heteroplasmy and copy numbers during long-term cultivation of mpTALEN-transfected heteroplasmic iPSCs. At 2 days post-transfection, cells were sorted and re-cultured for an additional 21 days (Figure 4A). The heteroplasmy shifting effects of the mp[vPKLB/PKR6C] were stronger than those of the hL-mp[vPKLB/PKR6C] at 2, 16, and 23 days following transfection (Figure 4B). The heteroplasmy levels in these mpTALEN-transfected iPSCs gradually decreased during 21 days of cultivation (Figure 4B). Additionally, we prepared a mutant mpTALEN, in which the *Fok*I nuclease domain was inactivated by the replacement of Asp450 with Ala.^22^ As expected, the inactivated version of the hL2-mpTALEN pair (hL2-mp[vPKLBm/PKR6Cm]) did not induce a heteroplasmy shift (Figure 4B). The cells transfected with the mutated hL2-mpTALENs showed the most moderate depletion of the mtDNA copy number on day 2 after the transfection (Figure 4C).

As reported in previous studies, the mtDNA depletion caused by mtDNA-targeted nucleases gradually recovered in the process of replication.^3^^,^^10^ The mtDNA copy number gradually increased in the hL-mp[vPKLB/PKR6C]-transfected cells during a 21-day period and finally reached a level close to that observed in untreated cells on day 2 (Figure 4C). The decline in the mtDNA copy number caused by the hL2-mp[vPKLBm/PKR6Cm] was recovered rapidly (Figure 4C). Contrarily, the recovery in the mtDNA copy number was suppressed in mp[vPKLB/PKR6C]-transfected cells (Figure 4C). These data indicated that the speed of mtDNA recovery is dependent on the mtDNA copy number on day 2 after the transfection. It was important to suppress the depletion in copy numbers as much as possible to promptly recover and expand mpTALEN-transfected iPSCs for subsequent experiments. To conclude, the hL-mp[vPKLB/PKR6C] was found to be the most appropriate G13513A-mpTALEN to decrease the m.13513G>A heteroplasmy level in MELAS-iPSCs.

### Generation of Doxycycline (Dox)-Inducible MYOD-Transfected MELAS-iPSCs (MyoD-iPSCs)

**Figure 5:**
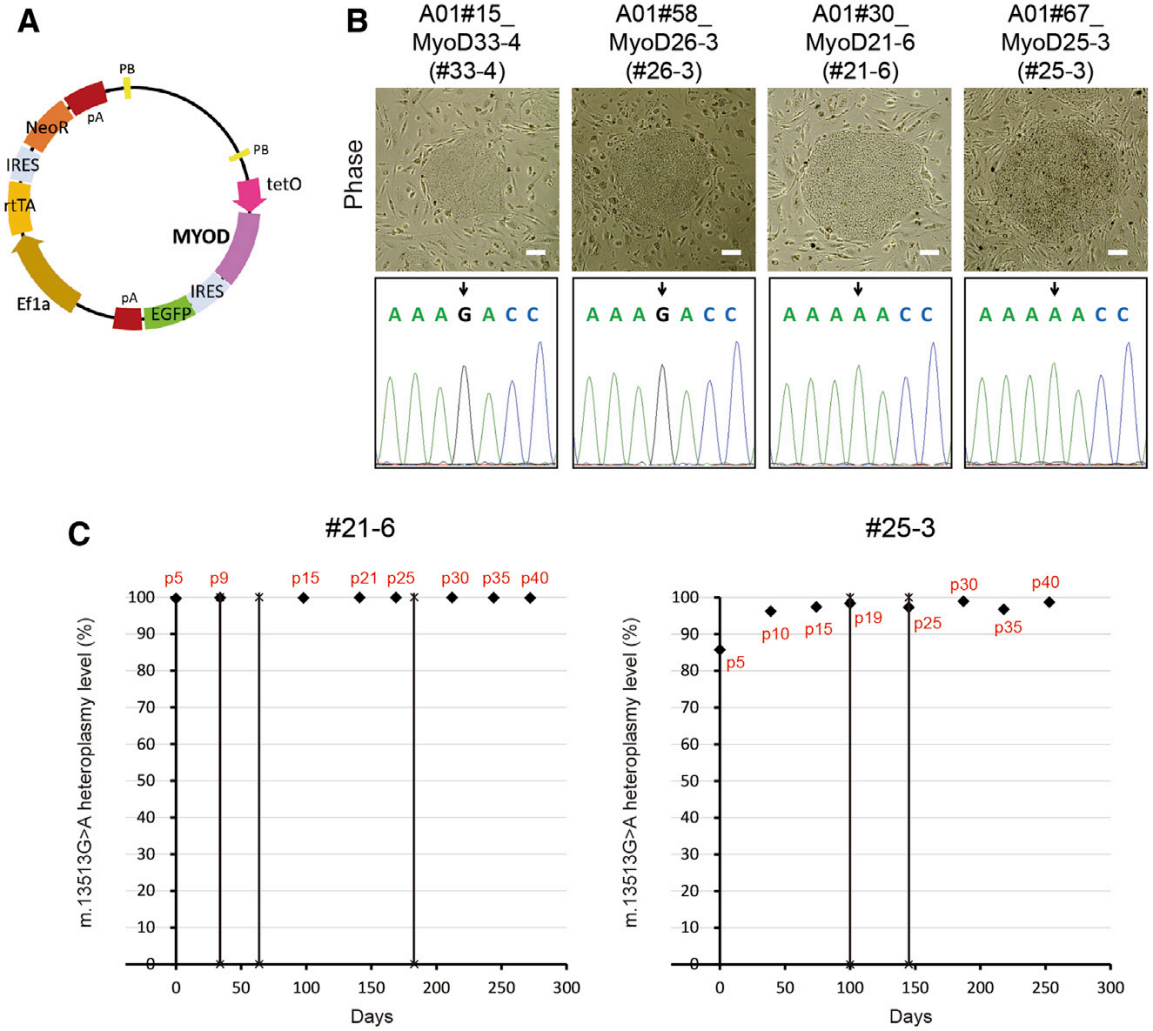
Generation and Characterization of MELAS-iPSCs with a Dox-Inducible Myogenic Differentiation System (MyoD-iPSCs) (A) Construction of the *piggyBac* vector for Dox-inducible MYOD expression (PB200_hMyoD). (B) Top: phase-contrast images of four MyoD-iPSC lines (A01 #15_MyoD33-4, #58_MyoD26-3, #30_MyoD21-6, and #67_MyoD25-3). Scale bars, 200 μm. Bottom: sequences of mtDNA extracted from the four MyoD-iPSC lines at passage 20. Arrows indicate the m.13513 position. (C) Genetic fluctuations of the m.13513G>A heteroplasmy levels in mutation-rich MyoD-iPSCs, #21-6 (left) and #25-3 (right), during long-term cultivation. Red letters next to diamonds indicate passage numbers. iPSCs were re-cultured after freezing and thawing at the time points indicated by vertical lines.

We tried to generate myocytes from MELAS-iPSCs as the first step of iPSC disease modeling. A transcription factor, *myogenic differentiation 1* (*MYOD*), is a master regulator of myogenic differentiation.^23^ We used a drug-inducible MYOD expression system to efficiently differentiate MELAS-iPSCs into myocytes. A tetracycline-inducible (Tet-on) human MYOD gene was introduced into previously established MELAS-iPSC lines (A01 #15, #58, #30, and #67)^8^ using the *piggyBac* vector (Figure 5A). The isolated MyoD-iPSC lines, named A01#15_MyoD33-4 (#33-4), A01#58_MyoD26-3 (#26-3), A01#30_MyoD21-6 (#21-6), and A01#67_MyoD25-3 (#25-3), expressed the pluripotency markers, OCT4, SSEA-4, and NANOG, which was confirmed by immunocytochemistry (Figure S7A). The expression of *OCT4* and *NANOG* was also confirmed by reverse-transcriptase (RT)-PCR (Figure S7B). The sequencing data indicated that the #33-4 and #26-3 lines were mutation-free (Figure 5B), while approximately 100% of mtDNA was mutant in the #21-6 line (Figures 5B and 5C). Meanwhile, the heteroplasmy level fluctuated in the #25-3 line (85.8% at passage 5) and gradually reached 100% during long-term cultivation (Figures 5B and 5C).

**Figure 6:**
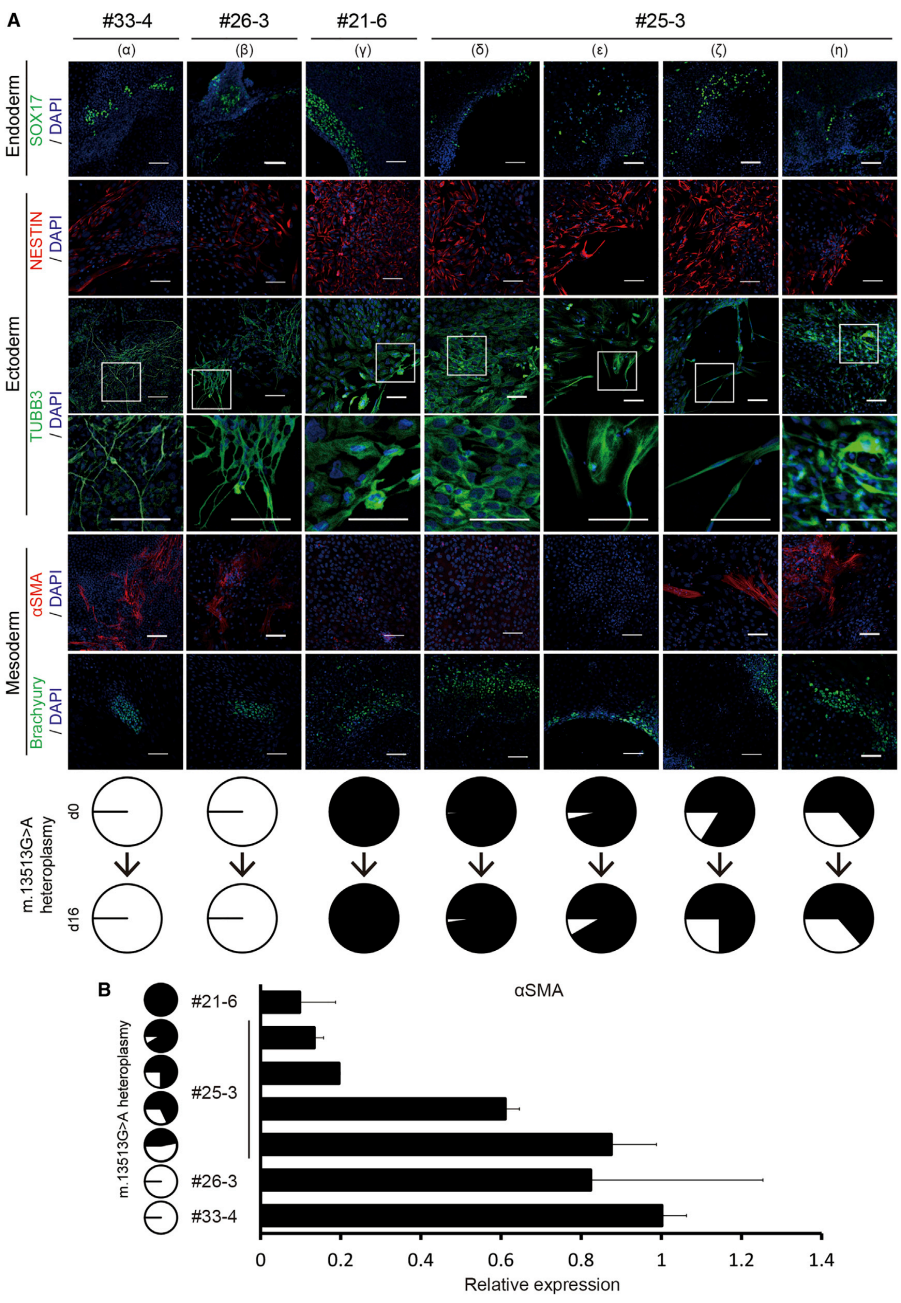
The Decrease in mtDNA Heteroplasmy Restores Spontaneous Differentiation Capacities of MELAS MyoD-iPSCs *In vitro* EB-mediated spontaneous differentiation of MyoD-iPSCs into all three germ layers are shown. (A) Differentiated cells from #33-4 (α), #26-3 (β), #21-6 (γ), and #25-3 (δ, ε, ζ, and η) were stained on day 16 with SOX17 (endoderm, green), NESTIN (ectoderm, red), TUBB3 (ectoderm, green), αSMA (mesoderm, red), and Brachyury (T) (mesoderm, green) antibodies. Nuclei were stained with DAPI. Scale bars, 100 μm. Pie charts at the bottom show m.13513G>A heteroplasmy in undifferentiated iPSCs on day 0 and in differentiated cells on day 16. Black and white regions represent percentages of mutant and wild-type mtDNA, respectively. (B) Quantitative RT-PCR analysis for *αSMA* in differentiated cells from four MyoD-iPSCs on day 16. The graph represents relative gene expression compared to the level of #33-4 (n = 3; error bars, SD). *ACTB* was used as internal control. Pie charts at the left side show m.13513G>A heteroplasmy levels in differentiated cells on day 16.

These selected iPSC lines formed embryoid bodies (EBs) after 8 days in floating culture. After an additional 8 days of differentiation in adherent culture, the expression of markers for three germ layers was analyzed by immunocytochemistry. SOX17 (endoderm)-, NESTIN (ectoderm)-, tubulin βIII (TUBB3) (ectoderm)-, and Brachyury (T) (mesoderm)-positive cells were detected in each culture (Figure 6A). These data also indicated that the four MyoD-iPSC lines retained pluripotency characteristics.

Contrarily, there were obvious differences between the mutation-free and mutation-rich iPSC lines. After switching to adherent culture, the spread of cells was slower from attached mutation-rich EBs than from mutation-free ones. The mutation-free iPSC lines (#33-4 and #26-3) could differentiate into α-smooth muscle actin (αSMA)-positive cells and TUBB3-positive neuronal cells after 16 days (Figure 6A, α and β). However, we could not find αSMA-positive cells in differentiated mutation-rich (≥98%) iPSC lines (#21-6 and #25-3) (Figure 6A, γ and δ), and there were many TUBB3-positive cells that did not extend neurites (Figure 6A, γ and δ) in three independent experiments, respectively. As mentioned in previous reports,^12^^,^^13^ these findings indicate that a high percentage of G13513A mutant mtDNA confers unusual differentiation properties to iPSCs.

To clarify the relationship between these unusual differentiation properties and G13513A mtDNA heteroplasmy in MELAS-iPSCs, we analyzed several varieties of a heteroplasmic iPSC line (#25-3) with a lower percentage of mutant mtDNA than 98%, which were prepared using G13513A-mpTALEN. The heteroplasmic #25-3 line with 96.1% of mutant mtDNA also showed unusual differentiation properties in EB-mediated spontaneous differentiation (Figure 6A, ε). However, the heteroplasmic #25-3 line, with 83.9% of mutant mtDNA, could differentiate into a few αSMA-positive cells and TUBB3-positive cells with a bipolar shape, which showed a heteroplasmy level of 75.0% on day 16 (Figure 6A, ζ). Quantitative RT-PCR data indicated that the αSMA expression level in differentiated cells with 75% mutant mtDNA from the #25-3 line was a little higher than those in mutation-rich (≥91.5% heteroplasmy) differentiated cells from the #21-6 and #25-3 lines (Figure 6B). Contrarily, differentiated cells with 68% heteroplasmy from the #25-3 line showed an about 60% αSMA expression level of those from the mutation-free #33-4 line (Figure 6B). In fact, it was easy to find αSMA-positive cells and TUBB3-positive neuronal cells in differentiated cells with 68% heteroplasmy or less (Figure 6A, η). These data demonstrated that MELAS-iPSCs with a high percentage of G13513A mutant mtDNA showed unusual differentiation properties, irrespective of the differences among iPSC clones in terms of their competence to differentiate into several somatic lineages.^24^

### Dox-Induced MYOD Overexpression Led to Efficient Differentiation of MyoD-iPSCs into Myocytes with or without Mutant mtDNA

**Figure 7:**
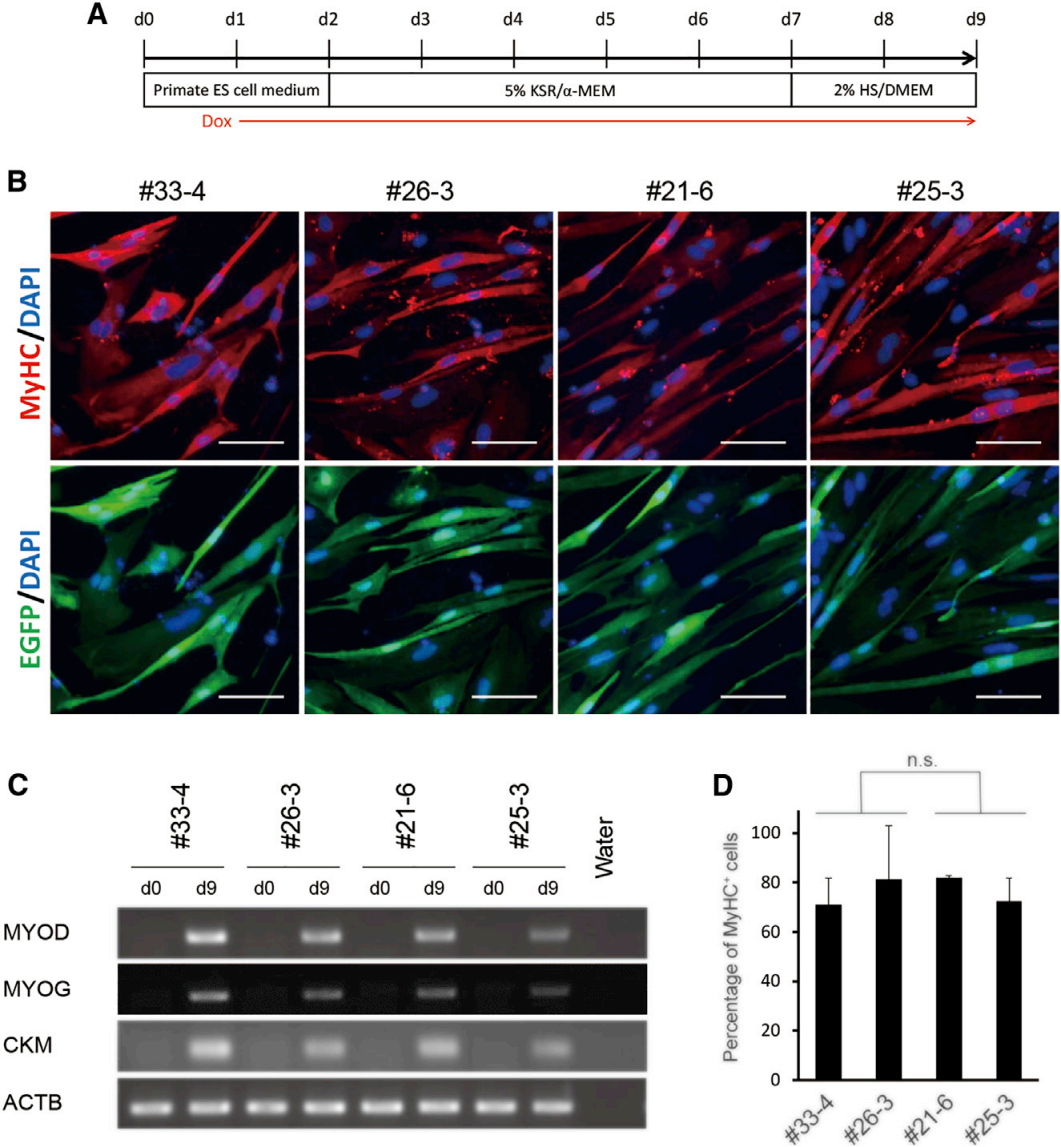
Characterization of Dox-Induced Myocytes from MELAS MyoD-iPSCs (A) Scheme of myogenic differentiation of MELAS MyoD-iPSCs. (B) Immunofluorescence of MyHC (red) in differentiated myocytes on day 9 and EGFP expression induced by Dox administration. Nuclei were stained with DAPI. Scale bars, 100 μm. (C) RT-PCR analysis of myogenic markers (*MYOD*, *MYOG*, and *CKM*) on day 0 (undifferentiated MyoD-iPSCs) and day 9 (differentiated cells). The ACTB gene served as an endogenous control. (D) Percentage of MyHC-positive cells differentiated from MyoD-iPSC lines on day 9. Data are expressed as the mean ± SD of three independent experiments; Student’s t test. n.s., not significant.

We next performed myogenic differentiation of MyoD-iPSCs through Dox-inducible MYOD overexpression, according to the schedule shown in Figure 7A. The Dox addition on day 1 of differentiation also induced EGFP expression (Figures 7B and S8). Transcripts of *MYOD*, as well as those of the transcriptional activator of myogenic differentiation *myogenin* (*MYOG*) and muscle cell-specific gene *creatine kinase*, *M-type* (*CKM*), were not detectable by RT-PCR on day 0 but were detected on day 9 of differentiation (Figure 7C). Differentiated cells on day 9 also showed much higher expression levels of *MYOD* compared to undifferentiated MyoD-iPSCs on day 0 as analyzed by quantitative RT-PCR (Figure S9). On day 9, differentiated cells from the four MyoD-iPSC lines were spindle-shaped and mostly positive for myosin heavy chain (MyHC), a marker of mature myocytes (Figures 7B and S8). Some dwarf binucleated myotubes were also observed (Figures 7B and S8). Unsurprisingly, m.13513G>A mtDNA was not detected in myocytes from the mutation-free MyoD-iPSC lines (#33-4 and #26-3). Conversely, the percentages of mutant mtDNA in myocytes induced from mutation-rich #21-6 and #25-3 MyoD-iPSCs were 100% and 99%, respectively (n = 3). The average percentages of MyHC-positive cells in differentiated cells on day 9 from all MyoD-iPSCs were higher than 70% (Figure 7D). There were no significant differences between the mutation-free and mutation-rich MyoD-iPSC lines in terms of the rate of MyHC-positive cells (Figure 7D). Additionally, percentages of MyHC-positive myocytes differentiated from heteroplasmic #25-3 iPSCs with low levels of mutant mtDNA (60% or less) were also similar to those of mutation-rich ones (Figure S10). These data demonstrated that this differentiation protocol could efficiently induce MyHC-positive myocytes from MELAS-iPSCs in a short period, with or without mutant mtDNA.

## Discussion

In this study, we refined a previously generated G13513A-mpTALEN by replacing an RVD at position 6 in R-mpTALEN(PKR) and by modifying codons at the N terminus. The new hL-mp[PKLB/PKR6C] showed a higher heteroplasmy shifting activity and a milder copy number depletion effect in heteroplasmic iPSCs with the m.13513G>A mutation. Unusual EB-mediated differentiation properties of mutation-rich iPSCs were recovered by decreasing the heteroplasmy levels using G13513A-mpTALEN. Furthermore, we demonstrated that iPSCs with drug-inducible MYOD expression (MyoD-iPSCs) could differentiate into MyHC-positive myocytes, with or without mutant mtDNA.

We developed a more effective G13513A-mpTALEN than the mp[PKLB/PKR] to reduce the proportion of mutant mtDNA in heteroplasmic iPSCs. Various types of pTALEN pairs were designed and evaluated using the SSA assay. Several G13513A-pTALEN pairs, including pTALEN(PKRX), pTALEN(PKRY), pTALEN(PKR4C), or pTALEN(PKR8C), showed a higher m.13513A(MUT) target specificity than did p[PKLB/PKR] but a much lower mtDNA-cleaving activity in the SSA assay. Modifications such as the elimination of the TALE module(s) or an introduction of an RVD–nucleotide mismatch in the TALE domain decreased the affinity between the TALEN monomer and the targeted DNA sequence, which resulted in a reduced cleavage activity of TALEN. However, the NI-to-HD RVD replacement at position 6 in pTALEN(PKR) did not result in an extensive reduction of DNA-cleaving activity.

Juillerat et al.^19^ have conducted a comprehensive analysis of TALEN activity and specificity using collections of TALE arrays containing all 64 possible RVD triplets (combinations of HD, NG, NI, and NN) at three defined consecutive positions. The study demonstrated that TALENs could only accommodate a relatively small number of position-dependent mismatches, while maintaining a detectable activity.^19^ In particular, the nuclease activity of one of the TALEN collections, which had the RVD pairs NN-NI-NN at positions 5-6-7 (corresponding to the DNA sequence GAG), similar to our pTALEN(PKR), was not decreased by introducing a mismatched RVD (NN-HD-NN), similar to our pTALEN(PKR6C). However, the relative nuclease activity of another TALEN, possessing the RVD pairs NG-NN-NN at positions 3-4-5 (corresponding to the DNA sequence TGG), which is also similar to our pTALEN(PKR), was decreased from 1 to 0.6 by introducing a mismatched RVD (NG-HD-NN), similar to our pTALEN(PKR4C). A similar trend was observed for the p[PKLB/PKR], p[PKLB/PKR4C], and p[PKLB/PKR6C] in our SSA assay. A crystallographic structural study of DNA-bound dHex3 (artificial TALE containing 11.5 TAL repeats)^25^ indicated that RVD loops in repeat 6 of molecule A and repeat 5 of molecule B exhibited an identical conformation, which was distinct from those in the other repeats. In these two repeats, the distance between Cα of Gly and the 5-methyl group of thymine is longer (>5 Å) than in the others (3.4–3.7 Å). The unusual structural features observed in the RVD loops of repeats 6/5 in the dHex3-DNA complex might explain the ability to tolerate an RVD-nucleotide mismatch in the pTALEN(PKR6C)-DNA complex. Fine-tuning of the conformational plasticity in the TALE domain by introduction of a nonconventional RVD might improve the nuclease activity and/or specificity of TALEN for point-mutated mtDNA.

Target specificity for m.13513G>A of p[PKLB/PKR6C] was higher than that of p[PKLB/PKR] as analyzed by the SSA assay. As could be expected based on these data, mp[PKLB/PKR6C] showed a higher activity in decreasing the percentage of mutant mtDNA than did mp[PKLB/PKR] in two heteroplasmic iPSC lines (#67 and #30). Additionally, cleaving activity of p[PKLB/PKR6C] against the wild-type sequence was also detected in the SSA assay (Figure 1B). Large depletion of mtDNA by mp[PKLB/PKR6C] application would be partially caused by off-target digestion of wild-type mtDNA. Contrarily, hL-mp[vPKLB/PKR6C] application did not induce large depletion of mtDNA, although it could change the heteroplasmy level. These data indicated that the cause of the mtDNA copy number depletion could not be explained by just the mtDNA-cleaving activity of mpTALEN. In other words, mpTALEN would have other unknown factor(s) to decrease mtDNA copy number depending on its expression level. Being able to identify other factors that reduce the mtDNA copy number, it could reveal improvements in applying mpTALENs more efficiently.

The application of the mp[vPKLZ/PKR8C] for 2 days could not induce a significant change in the mtDNA heteroplasmy, although the pair induced a decline in the mtDNA copy number. These data indicated that a pTALEN pair-based mpTALEN pair, whose nuclease activity was detected in the SSA assay, was not always sufficient to induce a significant heteroplasmic change in iPSCs within 2 days. In other words, mpTALEN pairs generated based on pTALEN pairs, whose SSA activities are lower than those of the p[PKLZ/PKR8C], are less likely to induce a significant heteroplasmic shift within a short period.

We demonstrated that a stepwise codon modification of mpTALENs (hL-mpTALEN and hL2-mpTALEN) could gradually reduce their expression levels. Furthermore, the reduction in the mpTALEN expression level by codon modification suppressed the depletion of mtDNA copy numbers in MELAS-iPSCs. However, the effect of G13513A-mpTALEN on mtDNA heteroplasmy was also decreased in a dose-dependent manner (Figures 3C, 4B, S5, and S6). A previous report regarding mtZFN for m.8993T>G mtDNA demonstrated that the reduction of the mtZFN expression, induced by the hammerhead ribozyme-based system, could not only suppress the depletion of the mtDNA copy number but it could also facilitate the heteroplasmy shift in cybrid cells.^10^ Further research is required to understand the effects of mutant mtDNA-targeted programmable nucleases on mtDNA profiles in terms of their DNA cleavage activity, target specificity, and expression level.

Application of the mp[vPKLB/PKR6C] showed the highest heteroplasmy-modifying effect after 23 days of cultivation. However, these mpTALEN-transfected iPSCs showed a lower proliferation rate in some cases. This feature is likely to be associated with a delayed repletion of the mtDNA copy number. However, the hL-mp[vPKLB/PKR6C] could induce a significant decline in mtDNA heteroplasmy on days 16 and 23 after the transfection while facilitating the repletion of the mtDNA copy number. This would be due to the activity of hL-mpTALEN, which was mildly sustaining but not inhibiting the recovery of mtDNA. Recent reports of adeno-associated virus (AAV)-mediated application of C5024T mtDNA-targeted programmable nucleases to a mouse model of heteroplasmic mitochondrial disease demonstrated a potentially universal route for treatment of mitochondrial diseases caused by mtDNA mutations.^26^^,^^27^ However, administration of high-dose mtZFN-AAV *in vivo* induced partial mtDNA copy number depletions.^26^ Fine-tuning mtDNA-targeted programmable nuclease levels by changing codons, such as our hL-mpTALEN, may contribute to improving its effects on mtDNA heteroplasmy and copy numbers irrespective of viral dose.

To understand the pathological mechanisms of mitochondrial diseases caused by mtDNA mutations, including MELAS, it is important to analyze cells differentiated from iPSCs of patients with mtDNA mutations. However, some reports have demonstrated that iPSCs from patients with mitochondrial diseases, who carry various types of mtDNA mutations, show differentiation defects *in vitro*.^14^^,^^15^^,^^28^ In this study, MyoD-iPSC lines (#21-6 and #25-3) with high proportions (≥96.1%) of G13513A mutant mtDNA showed poor differentiation into αSMA-positive cells and TUBB3-positive neuronal cells using a method of spontaneous EB-mediated differentiation. A previous study has demonstrated that iPSCs derived from patients with MELAS, who carried ∼100% of A3243G mutant mtDNA, could spontaneously differentiate into αSMA-positive cells and Tuj1-positive neuronal cells.^29^ These observations suggest that the heteroplasmic threshold required for iPSCs to show abnormal differentiation properties depends on the type of mtDNA mutation. Additionally, we succeeded in recovering unusual differentiation properties of G13513A heteroplasmic iPSCs by reducing their mutation load. These data also indicated that the unusual differentiation characteristics of #25-3 iPSCs were not due to their own differentiation-defective phenotype as for pluripotent stem cells.

During spontaneous differentiation of mutation-rich #25-3 iPSCs, the heteroplasmy levels were lower in differentiating cells on day 16 than in undifferentiated iPSCs on day 0. However, 63.7% of heteroplasmic iPSCs did not change the proportion of mutant mtDNA under the same differentiation condition (Figure 6A, η). It is difficult to determine the potential causes of these changes because multiple cellular processes modify heteroplasmy levels.^30^ If a higher percentage of G13513A mutation, than a given threshold, has a severe effect on replication of differentiating cells, then selection against this mutation will occur at the cellular level. This negative selection may influence the decrease of mutant mtDNA proportion in differentiating cells under this condition.

MyoD-iPSCs with a high proportion of G13513A mtDNA (≥99%) could differentiate into MyHC-positive myocytes upon Dox-induced MYOD overexpression. These MyoD-iPSCs would be preferable for disease modeling and *in vitro* cell-based drug screening because they can efficiently differentiate into myocytes within a short time, with or without mutant mtDNA. Direct induction via overexpression of a transcription factor may enable differentiation of iPSCs with a high proportion of mutant mtDNA to other cell types.

In summary, a fine-tuned G13513A-mpTALEN could contribute to the analysis of relationships between cellular phenotypes and the proportion of mutant mtDNA in isogenic iPSCs. Established MyoD-iPSCs, with or without mutant mtDNA, showed comparably efficient differentiation into myocytes. This study provides new insights into iPSC disease modeling and therapeutic development for mitochondrial diseases with mtDNA mutations, including MELAS.

## Materials and Methods

### Construction of TALEN Expression Plasmids

TALEN expression plasmids were constructed using the Platinum Gate TALEN kit^31^ (#1000000043; Addgene). We adapted a simple ligation method for four-module assembly and the Golden Gate method for final TALEN vector assembly using four-module ligand plasmids as described in the manufacturer’s protocol for the Platinum Gate TALEN kit. The destination vector used to express a pTALEN monomer with a +136/+63 scaffold under the control of the CAG promoter was modified to express an mpTALEN monomer. The modifications included the removal of the FLAG-tag and the nuclear localization signal, the inclusion of an MTS, derived from *ATP5B* or *COX8*, and an epitope tag [FLAG, V5,^20^ or HA] at the N terminus of the TALEN protein, as described in a previous report^8^ and in the Results. DNA fragments, including the MTS and epitope tag at the N terminus, were inserted into a destination vector, which was digested with *Hind*III and BamHI, using the In-Fusion HD cloning kit (Clontech Laboratories). Destination vectors for Lv-hL-mpTALEN, R-hL-mpTALEN, Lv-hL2-mpTALEN, and R-hL2-mpTALEN were generated using sequence-modified DNA fragments synthesized by GENEWIZ Japan (Saitama, Japan). Final hL-mpTALEN and hL2-mpTALEN plasmids were constructed using Golden Gate assembly. An expression vector of inactivated mpTALEN (point-mutated [D450A] in the *Fok*I domain^22^) was generated by fusing PCR-amplified DNA fragments and the restriction enzyme-digested original mpTALEN vector using the In-Fusion HD cloning kit (Clontech).

### SSA Assay Using HEK293T Cells

TALEN activity was evaluated using an SSA assay as previously described.^8^ The scheme of the SSA assay is shown in Figure S1A. HEK293T cells were transfected with two types of pTALEN plasmids, a reporter plasmid (pGL4-SSA-m.13513G(WT)-2 or pGL4-SSA-m.13513G(MUT)-2),^8^ and a reference plasmid for the dual-luciferase assay (pRL-CMV; Promega), using Lipofectamine LTX (Invitrogen). The dual-luciferase assay was performed 24 h following transfection using the Dual-Glo luciferase assay system (Promega) and an ARVO X5 luminometer (PerkinElmer) to evaluate the wild-type (m.13513G) or mutant (m.13513A) mtDNA-cleaving activity. The SSA activity (Luc/RLuc) was calculated as the ratio of firefly luminescence to *Renilla* luminescence. The m.13513A(MUT) target specificity of each pTALEN pair was calculated as [Luc/RLuc(MUT, pTALEN^+^) − Luc/RLuc(MUT, NC)]/[Luc/RLuc(WT, pTALEN^+^) − Luc/RLuc(WT, NC)], where NC is the negative control.

### Human iPSC Culture

All MELAS-iPSCs with the m.13513G>A mutation (A01 #15, #30, #58, and #67) used in this study were established as previously reported.^8^ These iPSCs were cultured on a mitomycin C-treated SNL feeder layer in primate embryonic stem cell (ESC) medium (ReproCELL, Japan) supplemented with 4 ng/mL basic fibroblast growth factor (bFGF) (FUJIFILM Wako Pure Chemical, Japan) and a 0.5% antibiotic-antimycotic solution (FUJIFILM Wako). These iPSCs were also cultured under feeder-free conditions, in accordance with a previous report.^32^ After the feeder cells were removed using a CTK solution,^33^ iPSCs were dissociated into single cells by incubation with 0.5× TrypLE Select (Gibco) for approximately 2 min at 37°C. Single iPSCs were reseeded on plates coated with iMatrix-511 (Matrixome, Japan) and cultured in StemFit AK02N medium (Ajinomoto, Japan) supplemented with 10 μM Y-27632 (FUJIFILM Wako).

### Cell Transfection and Sorting

MELAS-iPSCs were cultured under feeder-free conditions for the transfection of the mpTALEN plasmids. Single iPSCs were reseeded on dishes coated with iMatrix-511 (Matrixome) and cultured in StemFit AK02N medium (Ajinomoto) or primate ESC medium (ReproCELL) without bFGF. One day later, pCAGGS-EGFP^8^ and the mpTALEN plasmids (2 μg each) were introduced into iPSCs using Lipofectamine 3000 reagent (Invitrogen) according to the manufacturer’s protocol. Two days later, the cells were harvested and sorted using a MoFlo Astrios instrument (Beckman Coulter) with Summit acquisition software (Beckman Coulter) as previously described.^8^ A sorting gate was established based on the forward and side scatters, as well as on the level of EGFP expression, after the exclusion of dead cells and debris, which were stained with PI. EGFP-positive and PI-negative cells were directly sorted into StemFit AK02N or primate ESC medium with 10 μM Y-27632.

HEK293T and HeLa cells were transfected using Lipofectamine 3000 reagent according to the manufacturer’s protocol.

### Antibodies

The following primary antibodies were used: mouse anti-HA-tag (6E2) (immunocytochemistry [ICC], 1:100; western blot [WB], 1:1,000; #2367, Cell Signaling Technology), mouse anti-V5-tag (ICC, 1:300; WB, 1:1,000; R960-25, Invitrogen), rabbit anti-TOM20 (1:100; sc-11415, Santa Cruz Biotechnology), mouse anti-GAPDH (1:1,000; NB600-502, Novus Biologicals), mouse anti-SSEA-4 (1:100; MAB4304, Millipore), goat anti-NANOG (1:20; AF1997, R&D Systems), rabbit anti-OCT4 (1:200; ab19857, Abcam), mouse anti-αSMA (1:200; M0851, Dako), goat anti-Brachyury (1:20; AF2085, R&D Systems), goat anti-SOX17 (1:200; AF1924, R&D Systems), mouse anti-NESTIN (1:200; MAB5326, Millipore), rabbit anti-TUBB3 (1:1,500; PRB-435P, BioLegend), and mouse anti-MyHC (1:200; MAB4470, R&D Systems). Alexa Fluor 488 (A11055 or A11034, Molecular Probes)-, Alexa Fluor 594 (A11005, Molecular Probes; ab150132, Abcam)-, and Alexa Fluor 647 (ab150075, Abcam)-conjugated secondary antibodies were used for immunofluorescence study.

### Western Blotting

Transfected HEK293T cells were pelleted and lysed with a radioimmunoprecipitation assay (RIPA) buffer containing a protease inhibitor cocktail (Roche). After sonication and centrifugation, protein concentration of the supernatant was determined using a Pierce bicinchoninic acid (BCA) protein assay kit (Thermo Scientific). Equal amounts of protein samples were separated by SDS-PAGE on 4%–15% gels (Bio-Rad). Proteins were transferred to Immobilon-P membranes (Millipore). The membrane was blocked with 3% (w/v) skim milk in Tris-buffered saline with Tween 20 (TBS-T) and then incubated with primary antibodies at room temperature for 2 h, or at 4°C overnight. After washing with TBS-T, the membrane was further incubated with horseradish peroxidase (HRP)-conjugated anti-mouse immunoglobulin G (IgG) (GE Healthcare) at room temperature for 1 h. The membrane was washed with TBS-T, and protein signals were detected using the enhanced chemiluminescence (ECL) Prime western blot detection reagent (GE Healthcare). Images were analyzed using an ImageQuant LAS 4000 mini (GE Healthcare), and the band intensity was quantified using ImageJ. GAPDH served as a loading control.

### Generation of MyoD-iPSCs

We introduced a myogenic differentiation system^34^ into MELAS-iPSCs using a *piggyBac* vector, PB200_hMyoD,^35^ including (Tet-On)-MYOD-IRES-EGFP cDNA and a neomycin resistance gene. A01 MELAS-iPSCs were transfected with a transposase-expressing plasmid, pHL-EF1a-hcPBase^34^ (1.5 μg), and PB200-hMyoD (1.5 μg) using Nucleofector 2b (Lonza) and an Amaxa human stem cell Nucleofector kit 2 (Lonza), according to the manufacturer’s protocol, and were then plated on feeder cells. Forty-eight hours after the transfection, a G418 disulfate aqueous solution (50 μg/mL; Nacalai Tesque) was added to select appropriate MyoD-iPSC clones with high EGFP expression.

### Myogenic Differentiation of MyoD-iPSCs

Myogenic differentiation was performed according to a previous protocol,^34^ with some modifications. MyoD-iPSCs were dissociated into single cells by incubation with 0.5× TrypLE Select, and single cells were seeded on growth factor reduced Matrigel (Corning)-coated dishes without feeder cells. Matrigel was diluted 1:100 with primate ESC medium (ReproCELL), and the culture medium was changed to primate ESC medium with 10 μM Y-27632 but without bFGF. After 24 h, Dox (1 μg/mL; LKT Laboratories) was added to the culture medium. After an additional 24 h, the culture medium was changed to differentiation medium, composed of alpha minimum essential medium (α-MEM) (Nacalai Tesque) with 5% KnockOut Serum Replacement (KSR) (Gibco), a 0.5% antibiotic-antimycotic solution (FUJIFILM Wako), and 200 μM 2-mercaptoethanol (Nacalai Tesque). After an additional 5 days, the culture medium was changed to DMEM (high glucose; Nacalai Tesque) with 2% horse serum (Sigma), a 0.5% antibiotic-antimycotic solution, 2 mM GlutaMAX (Gibco), and 200 μM 2-mercaptoethanol.

### RNA Isolation and RT-PCR

Total RNA was purified using an RNeasy Plus mini kit (QIAGEN). One microgram of total RNA was used for an RT reaction with ReverTra Ace and a primer mix (Toyobo, Japan), according to the manufacturer’s protocol. PCR was performed with Ex Taq (Takara, Japan). PCR cycling conditions included initial denaturation at 95°C for 5 min, followed by 30 cycles of 95°C for 20 s and 60°C for 30 s. The primer sequences are shown in Table S1. PCR products were run on 1% agarose gel and stained with ethidium bromide.

### Quantitative RT-PCR

Quantitative RT-PCR was performed using an ABI Prism 7900HT instrument (Applied Biosystems) and GeneAce SYBR qPCR mix α (Nippon Gene, Japan). PCR cycling conditions included initial denaturation at 95°C for 10 min, followed by 45 cycles of 95°C for 30 s and 60°C for 1 min. ACTB was used as internal control. The primers used are listed in Table S1.

### EB-Mediated Spontaneous Differentiation of iPSCs

Spherical clusters of iPSCs re-suspended in DMEM/F12 (Gibco) containing 20% KSR (Gibco), 2 mM GlutaMAX (Gibco), 100 μM non-essential amino acid (NEAA) (Gibco), 100 μM 2-mercaptoethanol (Sigma), and a 0.5% antibiotic-antimycotic solution (FUJIFILM Wako) were transferred to Petri dishes. After an 8-day floating culture, spontaneously formed EBs were transferred to ECL (Millipore)-coated plates and incubated for another 8 days.

### Immunocytochemical Analysis

Cells were fixed with 4% paraformaldehyde in PBS for 30 min and then incubated in PBS containing 0.2% Triton X-100 for 10 min. Following blockade with 2% BSA in PBS for 1 h, cells were incubated with primary antibodies diluted with the blocking buffer and then washed with PBS. Finally, the cells were incubated with secondary antibodies, washed with PBS, and then mounted using ProLong Diamond antifade mountant with DAPI (Molecular Probes). Immunoreactive cells were visualized using an LSM 710 laser-scanning microscope (Carl Zeiss) and a Biorevo BZ-9000 fluorescence microscope (Keyence).

Myocytes differentiated from MELAS MyoD-iPSCs on a culture plate were stained with MyHC antibody and DAPI. Fluorescence of MyHC, EGFP, or DAPI was imaged using an Opera Phenix high-content screening system (PerkinElmer) at ×20 magnification and analyzed using Harmony software (PerkinElmer). The percentage of MyHC-positive cells was calculated as [MyHC^+^/EGFP^+^/DAPI^+^]/[EGFP^+^/DAPI^+^], segmented using “find cytoplasm” and “find nuclei” parameters.

### DNA Isolation

Genomic DNA, including mtDNA, was isolated from iPSCs and differentiated cells using a NucleoSpin tissue XS kit (Macherey-Nagel) according to the manufacturer’s protocol.

### Analysis of mtDNA Mutations

An mtDNA fragment, including the m.13513 position, was amplified using PrimeSTAR GXL DNA polymerase (Takara) with PCR primers (Mito-3F and Mito-3R; Table S1). The PCR amplicon was purified using the Wizard SV gel and PCR clean-up system (Promega) and Sanger sequenced using the ARMS_G13513_R1 primer (Table S1) by a sequencing service (FASMAC, Kanagawa, Japan).

### Analysis of mtDNA Heteroplasmy

Quantification of m.13513G>A mtDNA heteroplasmy was performed by ARMS-qPCR using an ABI Prism 7900HT instrument (Applied Biosystems), as previously described.^8^^,^^36^ The reaction mixture contained 0.3 ng of template DNA and GeneAce SYBR qPCR mix α (Nippon Gene), with primers ARMS-G13513_F1WT and ARMS-G13513_R1 for wild-type mtDNA species or ARMS-G13513_F1Mut and ARMS-G13513_R1 for mutant mtDNA species (Table S1). qPCR was performed in triplicate.

### Measurement of mtDNA Copy Numbers

mtDNA copy numbers in MELAS-iPSCs and iPSC-derived cells were determined as described previously.^8^ The reaction mixture contained 0.9 ng of template DNA and GeneAce SYBR qPCR mix α (Nippon Gene), with primers MT-CYB-F and MT-CYB-R for mtDNA or FBXO15-F and FBXO15-R for nuclear DNA (Table S1).

### Statistical Analysis

Statistical significance of differences was determined using a Student’s t test. Comparisons among three or four groups were performed using one-way ANOVA, followed by Tukey’s test (GraphPad Prism; GraphPad). Differences were considered significant at a p value <0.05.

## Supplemental Information

Document S1. Figures S1**–**S10 and Table S1

Document S2. Article plus Supplemental Information
