# Supplementary material for: Elimination of Mutant mtDNA by an Optimized mpTALEN Restores Differentiation Capacities of Heteroplasmic MELAS-iPSCs
Source: Mol Ther Methods Clin Dev. 2020 Oct 22;20:54–68. doi: 10.1016/j.omtm.2020.10.017 (PMC7744650; doi:10.1016/j.omtm.2020.10.017)
Supplement: Document S1. Figures S1–S10 and Table S1 [file mmc1.pdf]

OMTM, Volume *20*

Supplemental Information

Elimination of Mutant mtDNA by an Optimized mpTALEN Restores Differentiation Capacities of Heteroplasmic MELAS-iPSCs

Naoki Yahata, Hiroko Boda, and Ryuji Hata

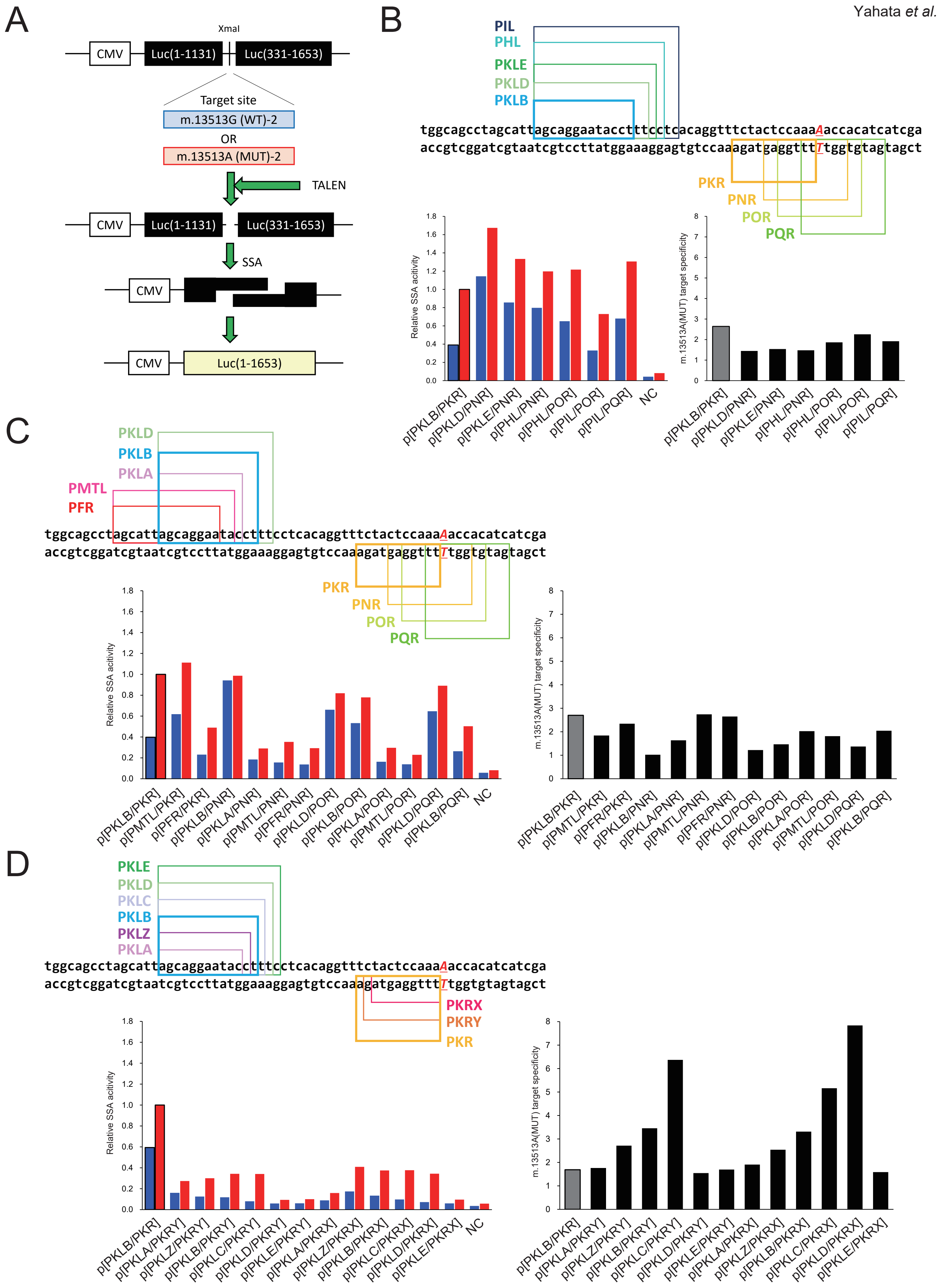

Supplementary Figure S1

Functional evaluation of engineered G13513A-pTALENs using an SSA assay.

(A) Scheme of the SSA assay. (B–D) Top: Schematic design of G13513A-pTALENs used in each assay. Colored boxes indicate RVDs in pTALEN monomers. TALE' s names are indicated next to the boxes. Left: Evaluation of relative SSA activity (Luc/RLuc) of pTALEN pairs (n = 1). Relative SSA activity is defined as the ratio of measured activity to the activity score of the p[PKLB/PKR]. NC, negative control. Right: m.13513A(MUT) target specificity of each pTALEN pair.

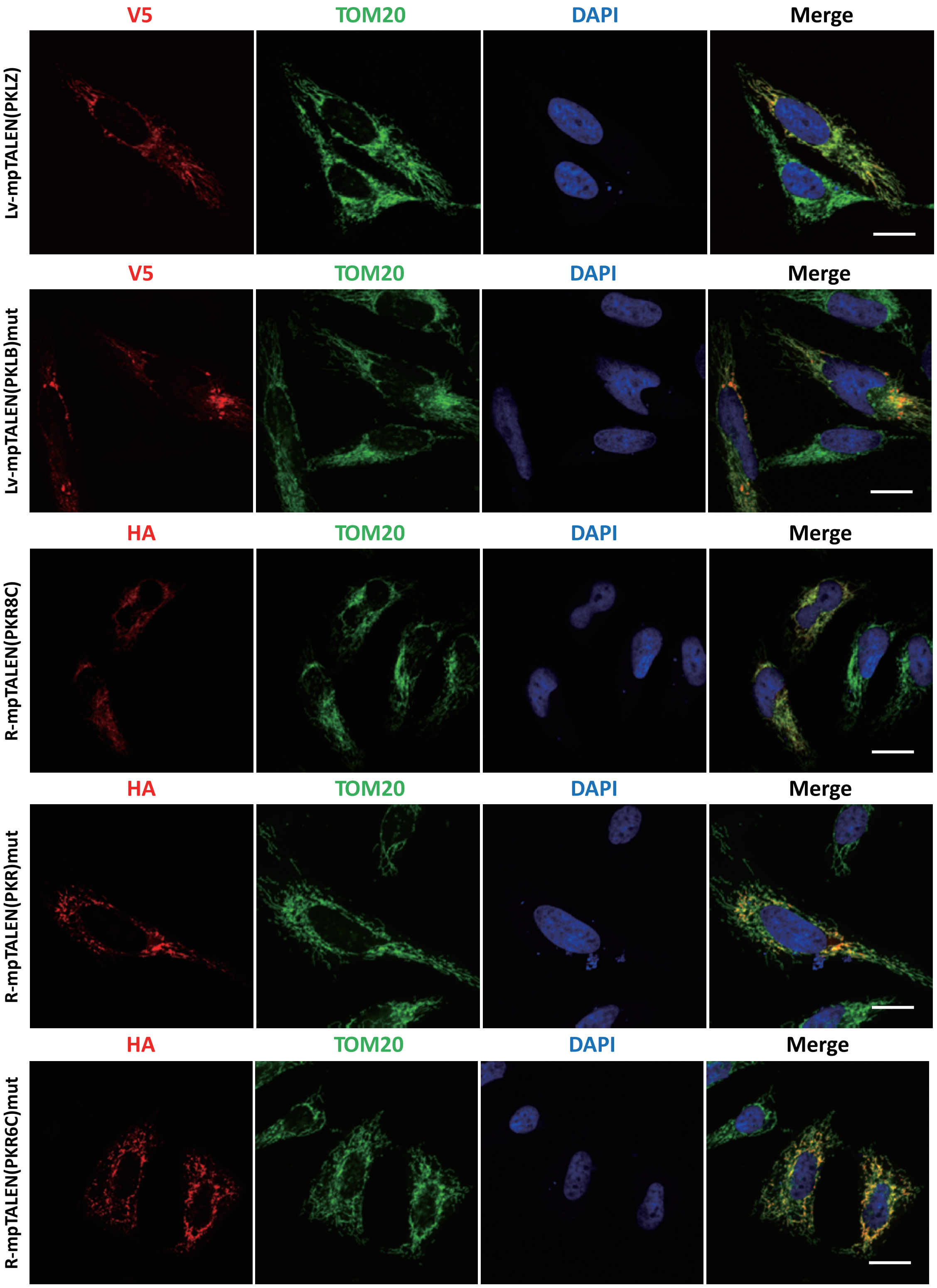

Supplementary Figure S2

Mitochondrial localization of mpTALENs, as analyzed by immunocytochemistry.

The mpTALEN monomer was transiently expressed in HeLa cells. Two days after the transfection, Lv-mpTALEN(PKLZ), Lv-mpTALEN(PKLB)mut, R-mpTALEN(PKR8C), R-mpTALEN(PKR)mut, and R-mpTALEN (PKR6C)mut were stained with an anti-V5 or anti-HA antibody (red). Mitochondria were stained with an anti-TOM20 antibody (green). Nuclei were stained with DAPI (blue). Scale bar, 20 μm.

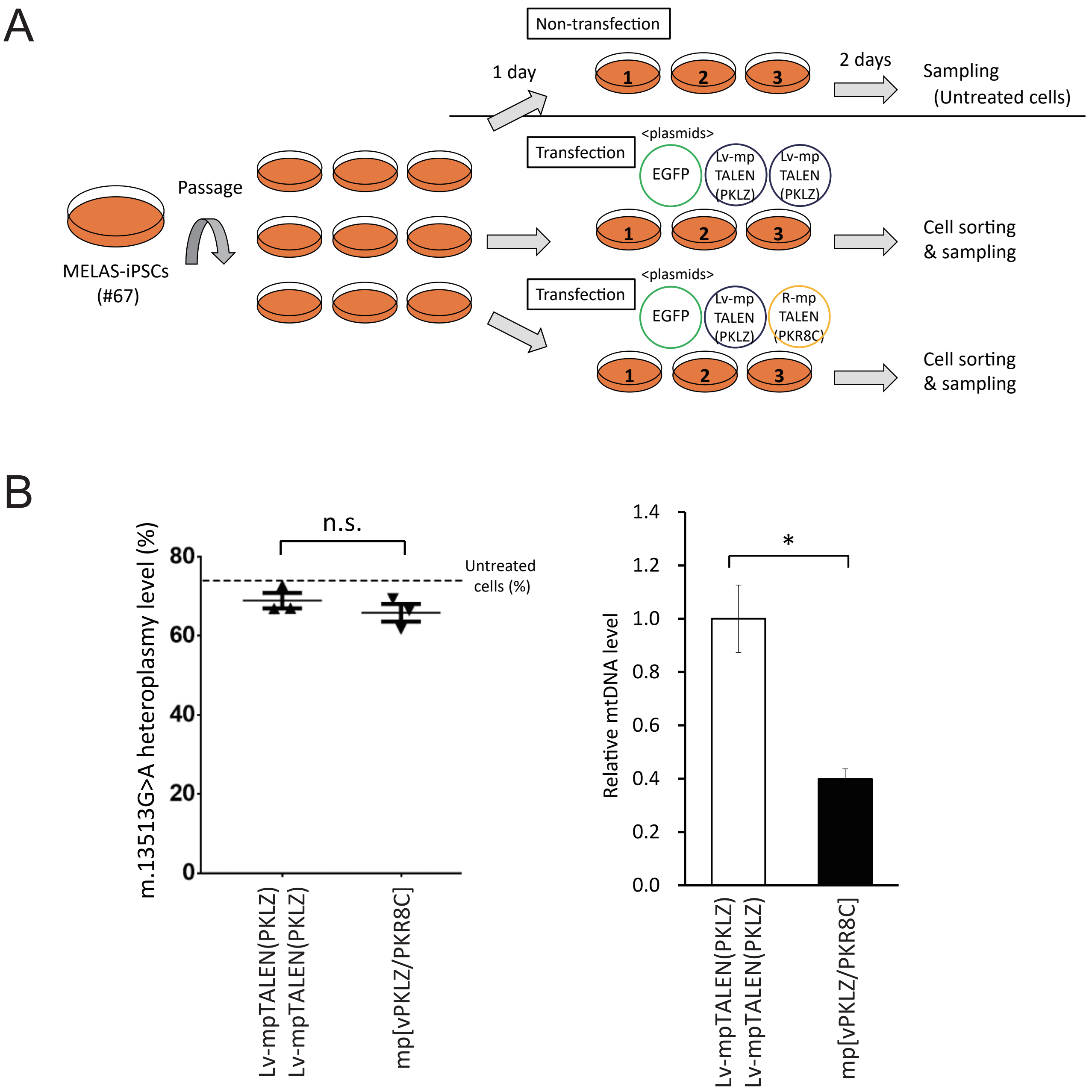

Supplementary Figure S3

Effect of the mp[vPKLZ/PKR8C] on the heteroplasmy level in #67-iPSCs on day 2 after transfection.

(A) Experimental scheme. MELAS-iPSCs (#67) were transfected at the same time with plasmids coding Lv-mpTALEN(PKLZ) and R-mpTALEN(PKR8C) and EGFP (n = 3). EGFP-positive and live cells were sorted on day 2 after the transfection and compared with sorted cells transfected with twice the amount of the plasmid coding Lv-mpTALEN(PKLZ).
(B, left) m.13513G>A heteroplasmy levels were analyzed using ARMS-qPCR. Dotted line indicates the heteroplasmy level in untreated cells. Data are expressed as the mean ± SEM (n = 3), Student' s *t*-test.
(B, right) mtDNA copy numbers in cells transfected with mp[vPKLZ/PKR8C] are presented relative to those in cells transfected with Lv-mpTALEN(PKLZ)/Lv-mpTALEN(PKLZ). Data are expressed as the mean ± SEM (n = 3). *$p < 0.05$ (Student' s *t*-test).

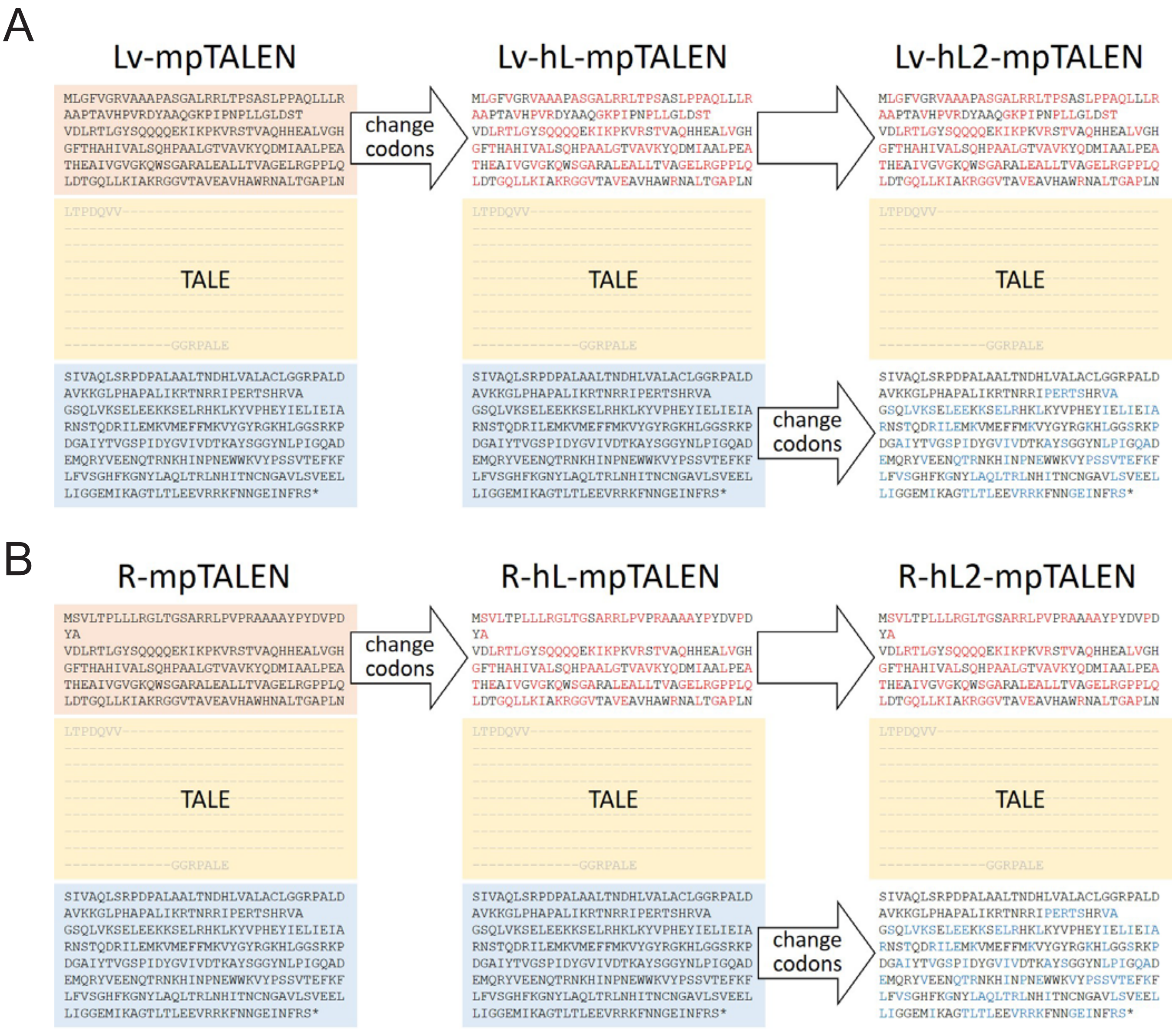

C

<Low-usage codons>

Ala(A): gcg	Pro(P): ccg
Glu(E): gaa	Gln(Q): caa
Gly(G): ggt	Arg（R): cgt, cga
Ile(I): ata	Ser(S): tcg
Lys(K): aaa	Thr(T): acg
Leu(L): cta, tta	Val(V): gta

Supplementary Figure S4

Codon modification in the Lv-mpTALEN and R-mpTALEN genes.

(A, B) The codons for N-terminal amino acids in the Lv-mpTALEN and R-mpTALEN genes, colored in red, were altered into selected codons (listed in [C]), with a lower frequency of usage in humans (resulting in Lv-mpTALEN and R-hL-mpTALEN, respectively). Furthermore, the codons of C-terminal amino acids in the Lv-hL-mpTALEN and R-hL-mpTALEN genes, colored in blue, were also altered into selected codons (resulting in Lv-hL2-mpTALEN and R-hL2-mpTALEN, respectively).

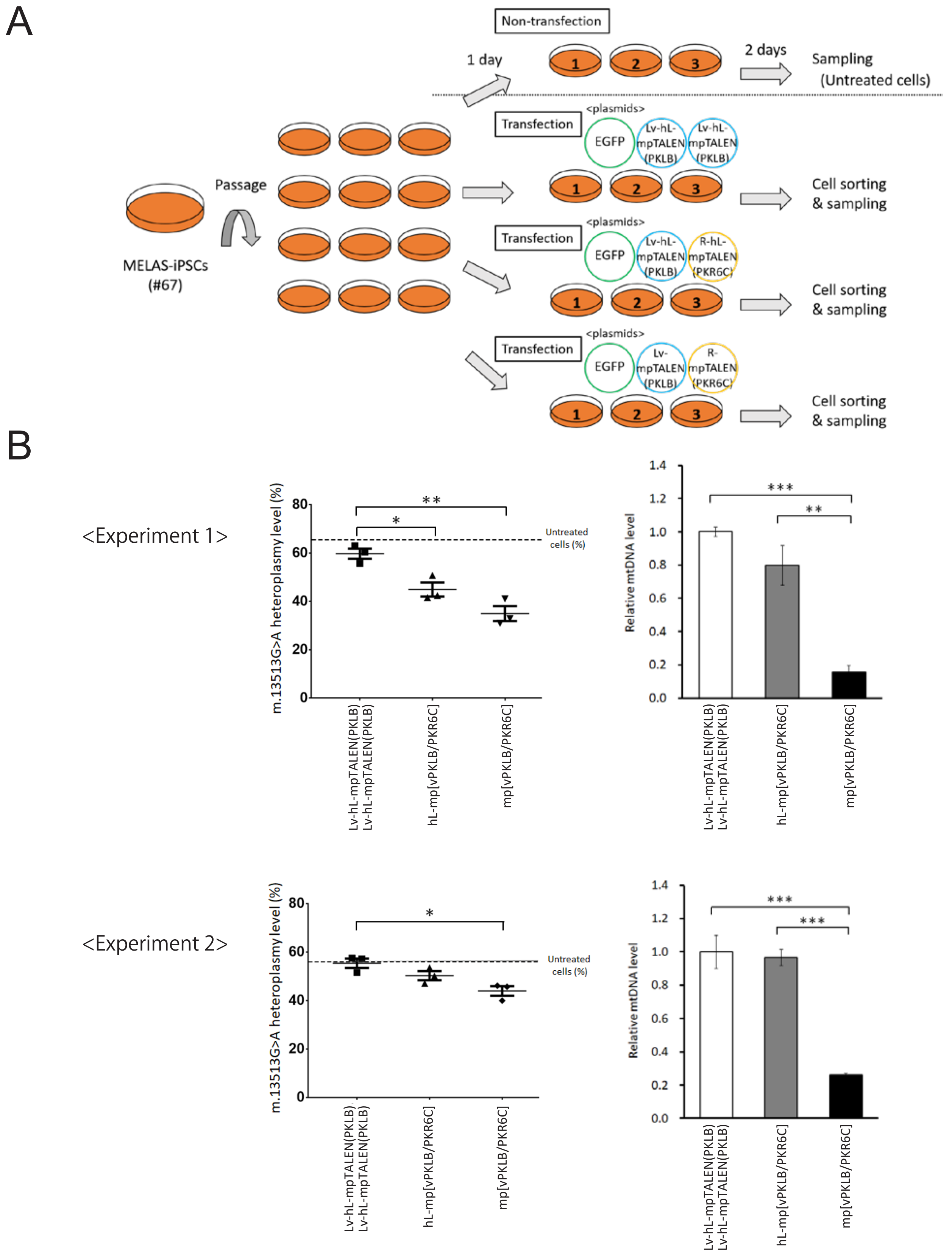

Supplementary Figure S5

Effect of the hL-mp[vPKLB/PKR6C] on the heteroplasmy level in #67 iPSCs on day 2 after transfection.

(A) Experimental scheme.

(B, left) m.13513G>A heteroplasmy levels were analyzed using ARMS-qPCR. Dotted line indicates the heteroplasmy level in untreated cells. Data are expressed as the mean ± SEM (n = 3). *$p < 0.05$, **$p < 0.01$ (Tukey' s test).

(B, right) mtDNA copy numbers are presented relative to those in cells transfected with Lv-hL-mpTALEN(PKLB)/Lv-hL-mp-TALEN(PKLB). Data are expressed as the mean ± SEM (n = 3). **$p < 0.01$, ***$p < 0.001$ (Tukey' s test).

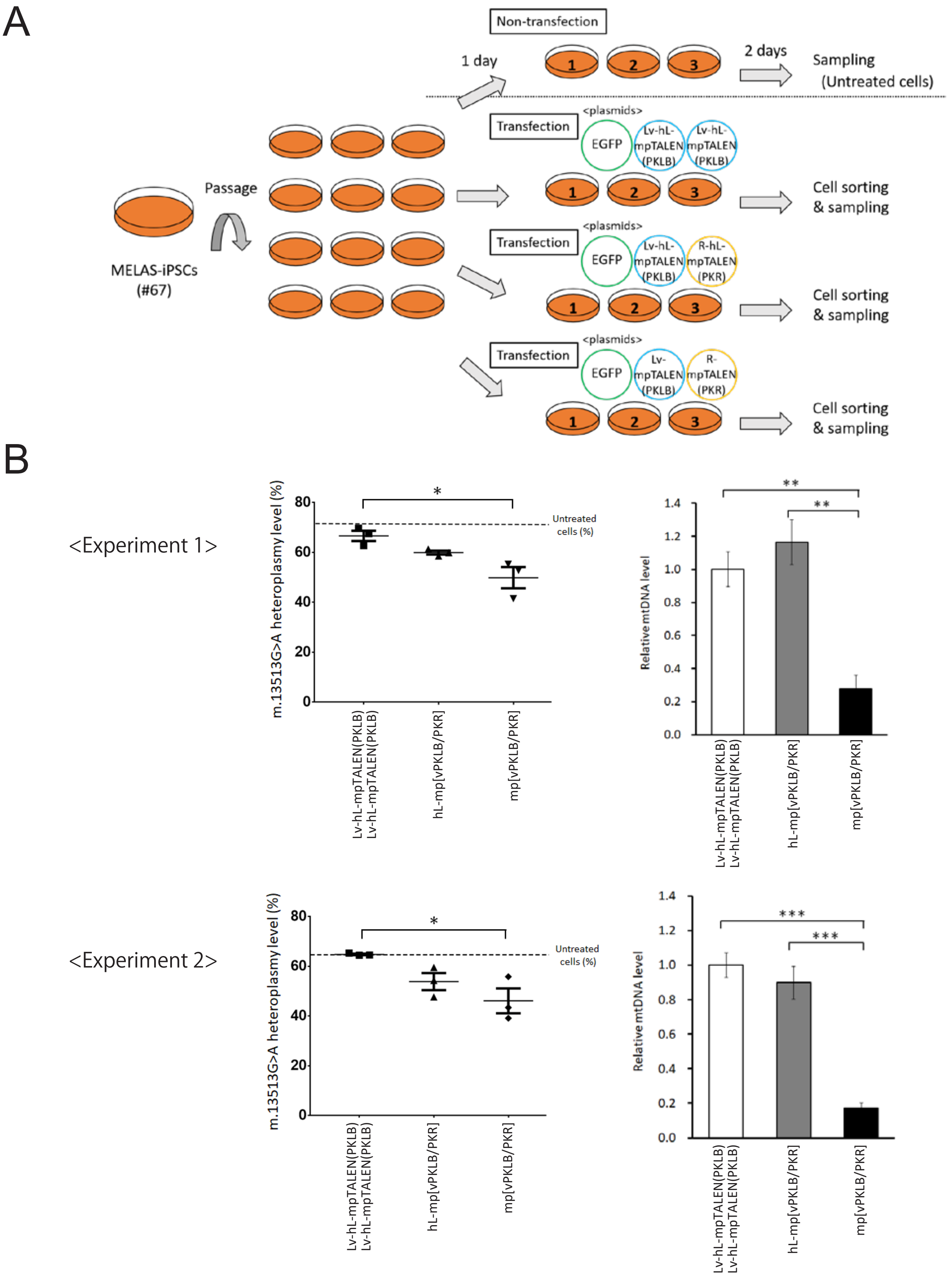

Supplementary Figure S6

Effect of the hL-mp[vPKLB/PKR] on the heteroplasmy level in #67 iPSCs on day 2 after transfection.

(A) Experimental scheme.

(B, left) m.13513G>A heteroplasmy levels were analyzed using ARMS-qPCR. Dotted line indicates the heteroplasmy level in untreated cells. Data are expressed as the mean ± SEM (n = 3). *$p < 0.05$ (Tukey' s test).

(B, right) mtDNA copy numbers are presented relative to those in cells transfected with Lv-hL-mpTALEN(PKLB)/Lv-hL-mpTALEN(PKLB). Data are expressed as the mean ± SEM (n = 3). **$p < 0.01$, ***$p < 0.001$ (Tukey' s test).

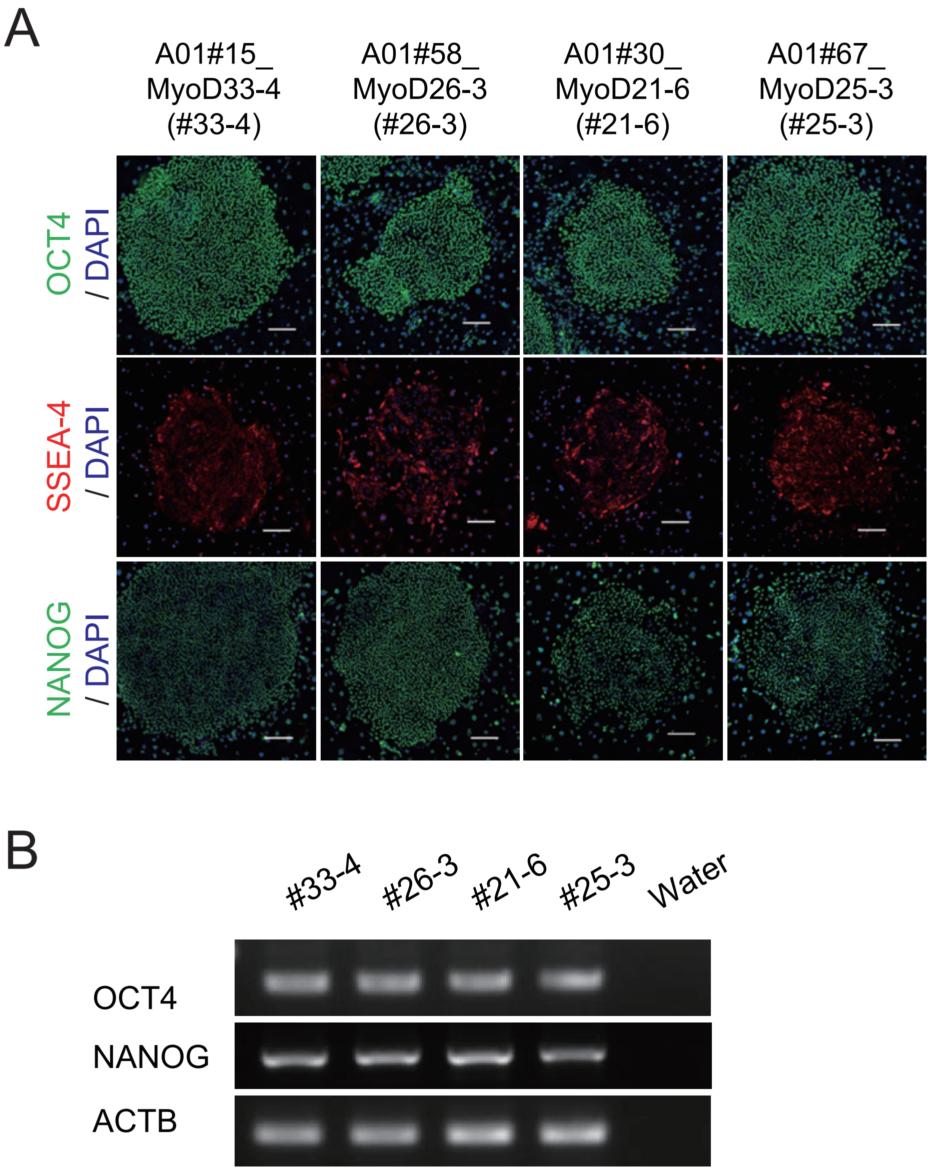

Supplementary Figure S7

Evaluation of pluripotency of MyoD-iPSC lines.

(A) Expression of pluripotency markers, OCT4 (green), SSEA-4 (red), and NANOG (green) in four MyoD-iPSC lines (A01 #15_MyoD33-4, #58_MyoD26-3, #67_MyoD25-3, and #30_MyoD21-6) were analyzed by immunocytochemistry. Nuclei were stained with DAPI. Scale bar, 200 µm.

(B) RT-PCR analysis of the four MyoD-iPSC lines for the expression of pluripotency markers (*OCT4* and *NANOG*). The ACTB gene served as an endogenous control.

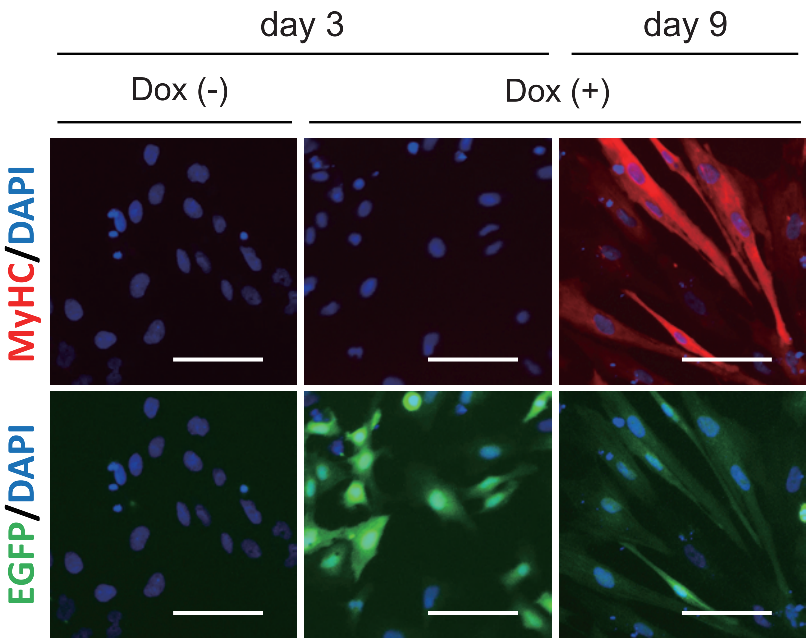

Supplementary Figure S8

The fluorescent cell images of myogenic differentiation in heteroplasmic A01#67-MyoD25-3 line.

Immunofluorescence of MyHC (red) was observed in differentiated myocytes from #25-3 line (47.9% heteroplasmy) on day 9 (right). Dox administration induced EGFP expression, which was observed on both days 3 (middle) and 9. Nuclei were stained with DAPI. Scale bar, 50 μm.

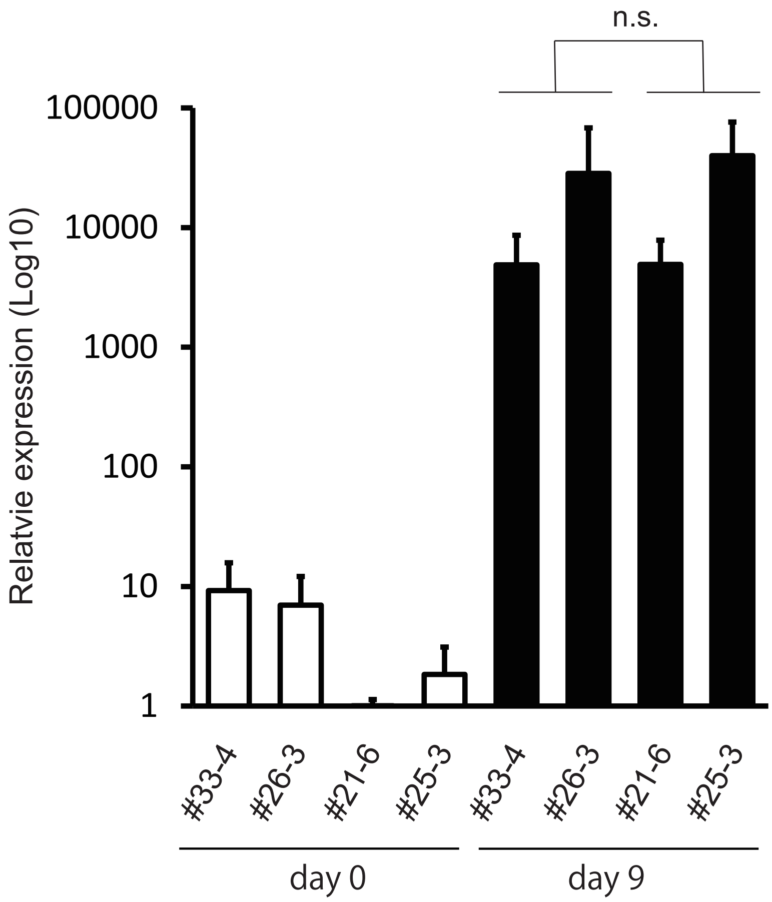

Supplementary Figure S9

Quantitative RT-PCR analysis for *MYOD* on days 0 and 9 of myogenic differentiation in four MyoD-iPSCs.

The graph logarithmically represents relative gene expression compared to the level of #21-6 at day 0 (n = 3; error bars, SD). *ACTB* was used as internal control. Student’ s *t*-test.

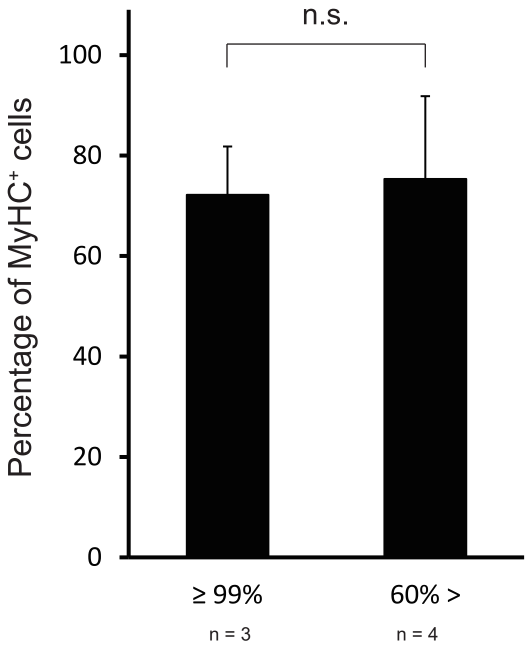

Supplementary Figure S10

Comparison between percentages of MyHC-positive cells on day 9 differentiated from #25-3 iPSCs showing high (≥ 99%) and low (60% >) levels of mutant mtDNA.

The error bars indiicate the SD. Student' s *t*-test.

Table S1: Primer list

**Name**	**Sequence**	**Name**	**Sequence**
Mito-3F	TCATTTTTATTGCCACAACTAACCTCCTCGGACTC	Mito-3R	CGTGATGTCTTATTTAAGGGGAACGTGTGGGCTAT
ARMS-G13513_F1WT	CTCACAGGTTTCTACTCCAATG	ARMS-G13513_R1	GACCTGTTAGGGTGAGAAGAA
ARMS-G13513_F1MUT	CTCACAGGTTTCTACTCCAATA		
MT-CYTB-F	TGCAACTATAGCAACAGCCTTCA	MT-CYTB-R	GAACTAGGTCTGTCCCAATGTATGG
FBXO15-F	GCCAGGAGGTCTTCGCTGTA	FBXO15-R	AATGCACGGCTAGGGTCAAA
MyoD-F	CACTCCGGTCCCAAATGTAG	MyoD-F	TTCCCTGTAGCACCACACAC
Myogenin-F	TGGGCGTGTAAGGTGTGTAA	Myogenin-R	CGATGTACTGGATGGCACTG
CKM-2nd-F	ACCTCAACCATGAAAACCTCA	CKM-2nd-R	GGCTGCTGAGCACGTAGTTA
ACTB-F	CCAACCGCGAGAAGATGA	ACTB-R	TCCATCACGATGCCAGTG
rt-Oct3/4-F	GACAGGGGGAGGGGAGGAGCTAGG	rt-Oct3/4-R	CTTCCCTCCAACCAGTTGCCCCAAAC
rt-Nanog-F	CAGCCCCGATTCTTCCACCAGTCCC	rt-Nanog-R	CGGAAGATTCCCAGTCGGGTTCACC
αSMA-fwd	CACTGCCTTGGTGTGTGACAAT	αSMA-rev	CGTAGCTGTCTTTTTGTCCCATTC
